# Automating wastewater characteristic parameter quantitation using neural architecture search in AutoML systems on spectral reflectance data

**DOI:** 10.1038/s41598-025-21069-4

**Published:** 2025-10-23

**Authors:** Shilpa Ankalaki

**Affiliations:** Manipal Institute of Technology Bengaluru, Manipal Academy of Higher Education, Manipal, India

**Keywords:** AutoML, Neural architecture space, Wastewater, Wastewater treatment plant, Neural network regression, Environment sustainability, Environmental sciences, Computer science

## Abstract

Wastewater (WW) analyses are required to establish optimal operation of treatment facilities, and rapid treatment assessment can greatly improve operational efficiency. WWTPs function through complex biochemical processes that exhibit high variability and are difficult to predict. This work presents the first in-depth study towards an original application of Auto Machine Learning (AutoML) models to accurately predict quality parameters of wastewater. This study aims to demonstrate the efficiency of neural network (NN) regression models built by applying the Neural Architectural Space (NAS) applying different search algorithms such as random search, grid search, Bayesian Optimization, and Hyperband search to predict the concentrations of various wastewater (WW) parameters such as biochemical oxygen demand (BOD), chemical oxygen demand (COD), ammonia (NH_3_-N), total dissolved solids (TDS), total alkalinity (TA), and total hardness (TH). The models are trained on data based on WW spectral reflectance data in the visible-near infrared range (400–2000 nm). The input variables consisted of spectral reflectance intensity of wastewater, while the output variables consisted of levels of six wastewater (WW) parameters, namely BOD, COD, NH_3_-N, TDS, TA, and TH. Different search algorithms were employed to identify the neural network (NN) architectures that provided single and multiple target variable (two to six target variables) predictions to the analysis. Experimental results indicate that the NN architecture optimized via Bayesian Optimization outperforms others, achieving the lowest mean absolute error (MAE) and a high coefficient of determination (R^2^). For single-target predictions on the validation set, the model attained R^2^ values of 0.9770 for BOD, 0.9860 for COD, 0.9800 for NH_3_-N, 0.9776 for TDS, 0.9847 for TA, and 0.9639 for TH. For two target predictions on the validation set, the model obtained R^2^ values of 0.988 for BOD and 0.993 for COD. Similarly R^2^ for predictions for three target variables are 0.961 for BOD, 0.962 for COD and 0.946 for NH3-N; for four target predictions 0.972 for BOD, 0.970 for COD, 0.955 for NH_3_-N and 0.972 for TDS; for five predictions 0.966 for BOD, 0.966 for COD, 0.951 for NH_3_-N, 0.966 for TDS and 0.966 for TA; and for six target predictions 0.987 for BOD, 0.99 for COD, 0.982 for NH_3_-N, 0.99 for TDS, 0.99 for TA and 0.718 for TH. Bayesian optimization search-based NN predicted the TH value with an R^2^ of 0.718. The Hyperband search NN obtained better results for TH prediction during six target predictions i.e. 0.9772 for BOD, 0.976 for COD, 0.975 for NH_3_-N, 0.976 for TDS, 0.976 for TA, and 0.899 for TH. This work automates the neural network architecture optimization process using NAS techniques. These methods systematically explore the hyperparameter space, removing the need for manual trial-and-error tuning across different datasets and target prediction tasks (both single and multi-target). The resulting neural network models are further interpreted using the LIME Explainable Artificial Intelligence (XAI) method.

## Introduction

Industrial wastewater and domestic sewage have been on the rise due to the increasing demand for water resources driven by rapid urbanization. Wastewater recycling may alleviate urban water demand and mitigate the degradation of water resources by providing a stable source of fresh water. Wastewater treatment plays a vital role in preventing the damaging consequences of human waste on ecosystems and protecting public health. Hence, wastewater treatment plants treat diverse streams of wastewater from industrial and municipal sources^1^.

It is key to environmental sustainability through alleviation of water scarcity and pollution^2^. To ensure that treatment processes remain within regulatory limits, water treatment facilities regularly monitor various wastewater parameters^3^. These include Biological Oxygen Demand (BOD), Total Hardness (TH), Total Alkalinity (TA), Total Dissolved Substances (TDS), Chemical Oxygen Demand (COD), and NH_3_-N.

BOD measures the amount of organic material in water that can be decomposed by anaerobic bacteria. TH is the sum of calcium and magnesium concentrations, expressed as mg/L of calcium carbonate. TA is measured by the amount of acid needed to bring the sample to pH 4.2 and is essential to maintaining pH balance during treatment. TDS represents the concentration of dissolved minerals, metals, salts, and organic matter. COD measures the oxygen required to chemically oxidize organic and inorganic substances. NH_3_-N, or free ammonia nitrogen, is toxic to microorganisms due to its ability to permeate cell membranes. To monitor wastewater, samples are collected from various areas of the treatment facility and examined using test kits or sent to laboratories. However, such procedures are time-consuming and may delay timely operational responses.

Wastewater treatment also offers the potential for renewable water generation^3^. As treatment must operate under strict regulatory conditions, water quality indicators like BOD, COD, NH_3_-N, TDS, TA, and TH are used to determine effluent quality^4^. Some tests, such as COD and BOD, take many hours or even days to complete, delaying decisions despite the presence of automated control systems^5]– [6^.

The use of Artificial Intelligence (AI) in wastewater treatment has gained significant traction over the past two decades. Machine Learning (ML), a branch of AI, uses statistical models and algorithms to detect patterns in data for making predictions. ML methods—such as artificial neural networks (ANN), random forests (RF), support vector machines (SVM), ensemble learning, and decision trees—have been applied in various domains including health, finance, and environmental studies^7–9^. For instance, researchers in^9^ used ML approaches to assess the effects of flood protection in Bangladesh.

Researchers utilized Multiple Linear Regression (MLR) and Support Vector Regression (SVR) to predict effluent concentrations of BOD, COD, NH₄-N, TN, and TP in both horizontal and vertical flow constructed wetlands (HFCWs and VFCWs)^10]– [11^. Analysis of 1232 datasets from 111 HFCWs revealed that media depth significantly influences redox conditions, microbial diversity, and pollutant removal rates. MLR and SVR models quantified relationships between depth and removal rate coefficients, while Grey Wolf Optimization identified optimal depths for maximum removal efficiency of various pollutants^12^.

Further optimization using 1680 datasets and five machine learning models (MLR, XGBoost, RF, ANN, and SVR) demonstrated that SVR achieved the highest accuracy in predicting first-order removal coefficients (k)^13^. Many studies have also evaluated ANN architectures for predicting wastewater characteristics. For instance, Fortela et al.^14^ used manual optimization of ANN architecture. However, manual tuning can be cumbersome and time-consuming.

All these works demonstrate the usage of ML algorithms for the prediction of various wastewater parameters but lack the automation of model optimization using AutoML and the application of Explainable AI (XAI) to interpret model results. This creates a research gap in leveraging AutoML for efficient model tuning and integrating XAI methods like LIME for enhancing model interpretability.

Based on this gap, the following research questions are framed:

- **RQ1**: How effectively can AutoML automate the search for optimal ML architectures in predicting wastewater quality parameters?
- **RQ2**: Which NAS algorithm yields the best trade-off between prediction accuracy and computational efficiency for the wastewater dataset?
- **RQ3**: To what extent does the integration of LIME improve the interpretability and transparency of the ANN model’s predictions?
- **RQ4**: What insights can LIME provide about the relationship between input wastewater features and their impact on the predicted outputs?

To address the above research questions, the proposed work conducts experiment on a dataset from Fortela et al., applying Neural Architecture Search (NAS) through AutoML methods to predict wastewater parameters.

The main goals and contributions of this work are:

1. **AutoML-based NAS Optimization**: Employed AutoML and NAS to automatically find the best configuration of the ANN, termed **RegNN-NAS**, which produces an optimal neural network architecture for regression using various NAS search algorithms.
2. **Wastewater Parameter Prediction**: Developed an advanced NAS-based ANN model for predicting wastewater characteristics—BOD, COD, NH_3_, TH, TA, and TDS—using different search strategies (Randomized Search, Grid Search, Bayesian Optimization, and Hyperband) to improve accuracy and reduce the burden of manual tuning.
3. **Explainability via XAI**: Integrated Explainable AI techniques, specifically LIME, to enhance the interpretability of ANN predictions. LIME helps make the model outputs transparent and user-friendly by visually showing the influence of input features on the predicted outputs.

## Related work

Hyperspectral methods have been increasingly utilized for monitoring natural resources in recent years. They reduce interference from the surrounding environment, give high-resolution spectral information, and increase the precision of spectral measurements. In recent years, hyperspectral technology has been utilized for estimating pollutant concentrations, focusing on the VIS-NIR bands^15^. At present, water quality inversion methods mainly concentrate on physiological indicators (transparency, nutrient content, etc.)^16^, whereas they pay relatively less attention to biochemical indicators such as TH, TDS, BOD, COD^17^, TA, NH_3_-N^18^. Water quality parameters serve as important indicators of pollution, playing a crucial role in guiding the effective treatment of wastewater. Among the different methods for spectral inversion of these parameters, empirical statistical approaches remain the most commonly used^19^.

In a study conducted at a large municipal wastewater treatment plant in KwaZulu-Natal, South Africa, researchers compared seven adaptive and ML models—including LSTM, BiLSTM, SVR, and ANN—to predict water quality parameters. Among these, the BiLSTM model consistently outperformed the others, recording error rates between 3.1% and 9.8% and achieving low mean absolute errors for COD, ammonia, pH, and TSS. Although the JITTD model also showed promising results for several parameters, it had difficulties with soluble reactive phosphate prediction. These findings highlight the potential of ML-based soft sensors, especially BiLSTM, to enhance monitoring and optimization of wastewater treatment processes^20^.

Researchers of^21^ employed ML to forecast wastewater quality in two distinct environments—a simulated plant (by DHI WEST software) and a real plant (Santa Catarina Brewery WWTP, Brazil). Suitable models used to test these scenarios were based on RF, SVM, and a Multi-Layer Perceptron (MLP). In particular, the MLP model reached an R^2^ of 0.72 on total nitrogen (TN), but again only for the simulated test set, while on the real data RF was superior despite some variation. Importances from feature importance analyses (using PDP and Permutation Importance) identified predominant nitrogen parameters as leading predictors. High-quality data and operational details were highlighted as critical parameters to improve predictive performance in WWTPs.

In^22^, researchers created an algorithm for data collection and analysis processes at wastewater treatment plants. They worked on regression tasks to predict the quality of treated wastewater and classification tasks to prevent emergencies, with a focus on filamentous bulking in activated sludge. They treated laboratory-simulated data as the training set and a smaller dataset from real treatment facilities as a test set. Best results were reached by gradient-boosting models from the CatBoost family with SMAPE = 9.1 for regression and ROC-AUC = 1.0 for classification. The study also revealed the key predictors for accurate modelling.

Wang^23^ discussed the effect of operational factors on removal of suspended solids and orthophosphate at Umeå wastewater treatment plant, Sweden with more than 105,763 data points (32 predictors), the study used a Variable Importance Measure (VIM) analysis and faced limitations of being a “black box” model. The authors highlighted the importance of addressing treatment process time lags. Xu^24^ also built an LSTM model to predict treatment quality based on low-cost sensor data considering time delays.

According to the authors of^25^, Neural Network (NN) regression models could predict wastewater quality parameters such as BOD, COD, NH3-N, TDS, TA, and TH using spectral reflectance data (bands close to 400–2000 nm). High R^2^ (0.994 for training, 0.973 for testing) and low MAE values were obtained based on single-variable output models rather than multi-variable models. A grid search of hyperparameters combined with k-fold cross-validation was performed to improve performance, underscoring the potential of NNs in the analysis and treatment of wastewater. The applications of NN and other models are also used in the applications of seawater biodegradability^26–28^. Researchers of^29^ demonstrated the ability of high-end Raman spectroscopy to decouple complex mixtures, a concept that can also be applied to wastewater reflectance analysis. Their approach emphasizes the need for signal intensity and feature-dense spectra, which encourages our application of spectral data in AutoML frameworks. Researchers in^30^ used light-weight NAS models to maximize signal processing under energy constraints. Although their deployment was focused on microwave-based dual-function systems, the underlying aspects of efficient architecture searching and signal interpretation remain relevant to automated, field-deployable wastewater monitoring systems. Hu et al.^31^ developed trustworthy multi-focus fusion framework for sewer defect detection, highlighting the utility of reliable, multi-label deep learning. Their contributions demonstrate the utility of explainable and resilient AutoML frameworks for wastewater applications, particularly when measuring numerous chemical parameters from spectral data.

Liu et al.^32^ emphasized worldwide threats of polluted water and the urgent need for data-driven decision support systems. Our work adds to this agenda through automating wastewater quantitation with scalable AutoML systems. Table 1 depicts the state-of-the-art ML models employed for wastewater parameters.

**Table 1:** State-of-the-art ML models employed for wastewater parameters.

Method type	Dataset/context	Model	Parameter (s)	MAE	RMSE	*R*^2^	Limitations/notes	Reference
Support vector machines	Real wastewater treatment plant (AMBEV WWTP)	SVM	COD	29.94	30.86	− 11.97	Auto optimization of ML hyperparameters	^21^
	VFCW	SVR	NH_4_-N		1.35	0.89	XAI approaches	^10^
			TN		3.64	0.90		
			TP		0.08	0.87		
	Real-time South African municipal WWTP	SVR	Conductivity (Con)	8.35	50.47	–	XAI approaches for interpretation	^20^
			COD	21.04	24.86	–		
			TSS	12.16	16.22	–		
Neural networks (ANN, LSTM, BiLSTM, DNN)	Real-time South African municipal WWTP	LSTM	Conductivity (Con)	2.28	50.21	–	XAI approaches for interpretation	^20^
		BiLSTM	COD	1.54	24.89	–		
	Umeå WWTP	DNN	Phosphate in effluent (PO4e)	–	–	0.872	Incorporating ML interpretation frameworks	^23^
	Lab-scale two-staged A/O WWTP	LSTM	Influent NH_4_-N	–	0.101	–	Auto optimization of ML hyperparameters	^24^
	The spectral reflectance dataset	ANN (multiple targets)	Influent BOD	2.415	–	0.973	Auto optimization of NN hyperparameters	^14^
Tree-based models (RF, CatBoost)	Real wastewater treatment plant (AMBEV WWTP)	RF	COD	30.03	30.95	− 3.30	Auto optimization of ML hyperparameters	^21^
	Moscoe Region, Russia WWTP	CatBoostRegressor	BOD (Effluent)	0.670	0.962	0.78		^22^
			COD (Effluent)	0.854	1.211	0.85		
			Ammonium (Effluent)	0.111	0.145	0.79		
			Phosphorus (Effluent)	0.036	0.054	0.81		
			Nitrate (Effluent)	0.484	0.765	0.82		
			Nitrite (Effluent)	0.003	0.006	0.88		
	Umeå WWTP	RF	PO4e	–	–	0.886	Incorporating ML model interpretation systems to enhance the framework and improve the reliability of its results.	^23^
			TSSe	–	–	0.920		
Regression models (MLR, rPCA-MARS)	Lab-scale two-staged A/O WWTP	MLR	Influent NH_4_-N	–	0.203	–		^24^
	The spectral reflectance dataset	Linear rPCA-MARS	BOD	9.879 (MSE)	–	0.959	XAI approaches for interpretation	^25^
			COD	213.072 (MSE)	–	0.967		
			NH_3_-N	3.032 (MSE)	–	0.936		
		Cubic rPCA-MARS	BOD	12.508 (MSE)	–	0.939		
			COD	284.445 (MSE)	–	0.945		
			NH_3_-N	3.113 (MSE)	–	0.934		

Existing research lacks explainability (XAI), AutoML optimization, and model generalization across different WWTPs. To overcome this, the current work proposed the RegNN -NAS, which optimizes the neural network for regression task using various search algorithm of NAS.

## Proposed methodology

**Fig. 1:**
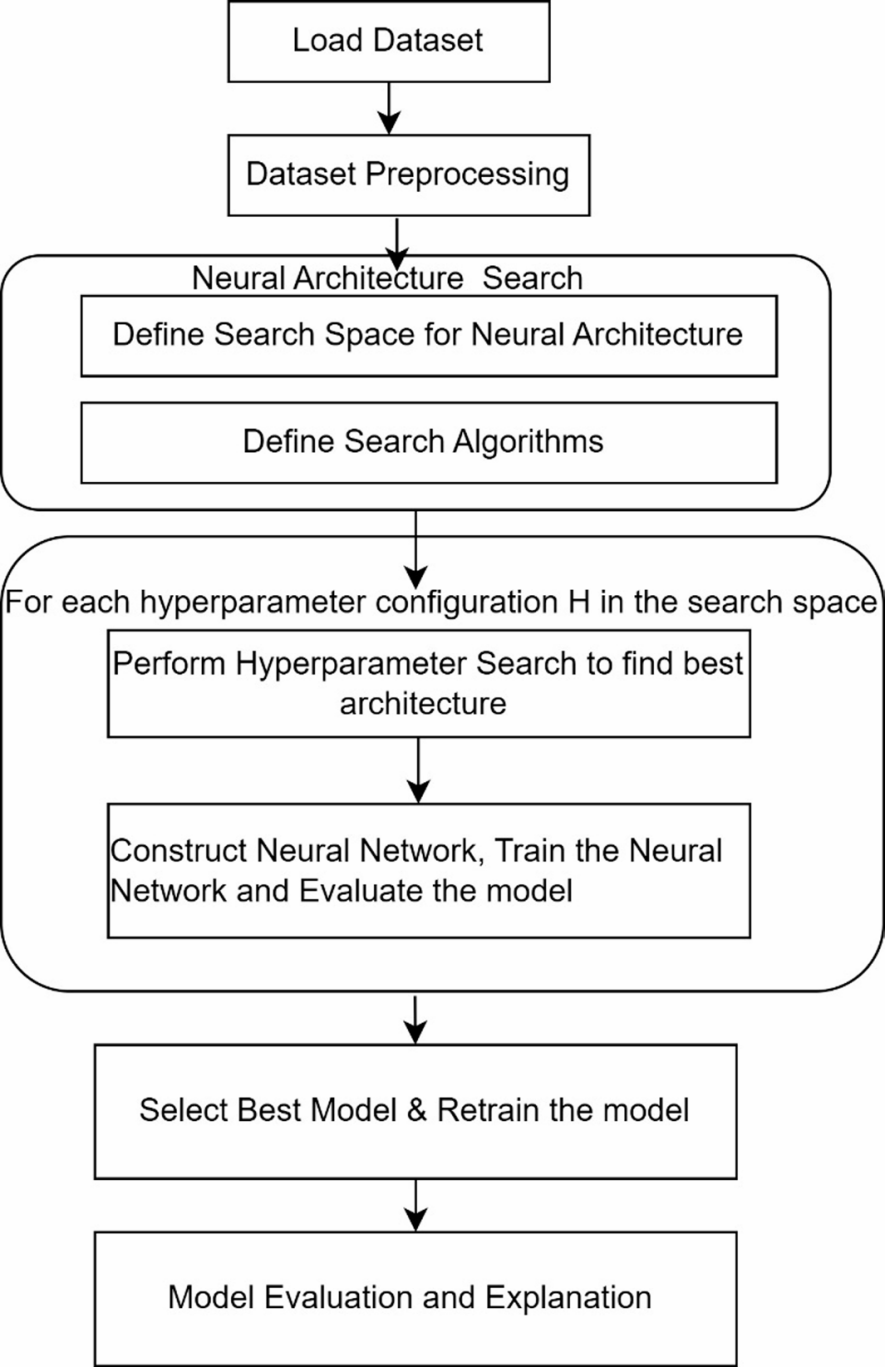
Flow of proposed methodology.

The proposed methodology includes the NAS to search for an optimized neural network. Figure 1 depicts the steps involved in the proposed methodology. This work utilized four search algorithms, namely Random search, Grid search, Bayesian Optimization and the Hyperband Algorithm. The proposed work utilized the spectral reflectance dataset to predict the values of BOD, COD, NH_3_-N, TDS, TA and TH. The WW spectral reflectance data in the visible to near-infrared spectrum (400–2000 nm) was originally collected by^19^. The proposed methodology employs Artificial Neural Networks (ANNs) due to their proven capability to model complex nonlinear relationships effectively. As summarized in Table 1, previous studies have demonstrated that ANNs consistently outperform other models in predicting wastewater parameters. For instance, the study in^14^ conducted extensive experiments on the dataset originally collected by^19^ and concluded that ANN models were well-suited for this prediction task. However, their approach relied on manual, trial-and-error-based hyperparameter tuning, which may lead to suboptimal model performance. To address this limitation, the present study reuses the same dataset but enhances the modeling process by leveraging Neural Architecture Search (NAS) within an AutoML framework to automatically optimize the ANN architecture and hyperparameters.

### Dataset collection

In total, water samples of WW were taken from the inlet (influent WW), anoxic tank, aerobic tank, sedimentation tank, and outlet (effluent WW) of a municipal wastewater treatment facility. These were collected from different treatment sites at a local sewage treatment plant^19^. The chemicals of the samples have been analysed thoroughly^19^. Two groups of the dataset were found according to the quality of wastewater, Group 1 the influent WW with COD, BOD and NH3-N value while Group 2 highest in the anoxic tank, aerobic tank, sedimentation tank and finally effluent WW concerning the three parameters. It consists of spectral reflectance data of the wastewater in the range of 400–2000 nm (visible to near-infrared region), along with BOD, COD, NH3-N, TDS, TA, and TH concentrations. These were treated as target variables during the learning phase. The input data consisted of banded spectral reflectance values measured at wavelengths ranging from 400 nm to 2000 nm, with each band serving as a feature. Each input sample was represented as a vector comprising 1,601 features.

### Data preprocessing

During modeling, the dataset was partitioned into training and testing subsets based on the specific dataset used. When working with the entire dataset, a 90:10 split was applied, allocating 90% for training and 10% for testing. Similarly, for the Influent WW dataset (Group 1), the same 90:10 partitioning was used.

To ensure all features were on a comparable scale and to improve the stability of the model training process, Min-Max Normalization was applied to the input data. The MinMaxScaler from the Scikit-learn library was used, which transforms each feature to a fixed range of [0, 1] based on Eq. 1:

1 $$\:{X}^{{\prime\:}}=\frac{X-{X}_{min}}{{X}_{max}-{X}_{min}}$$

where, X represents the original feature matrix, Xmin and Xmax denote the minimum and maximum values of each feature within the training dataset, and X′ is the scaled feature matrix.

To prevent data leakage, the normalization parameters (Xmin and Xmax) were computed exclusively from the training set. These parameters were then used to transform both the training and validation datasets as shown in Eqs. 2 and 3.

2 $$\:{X}_{train}^{{\prime\:}}=\frac{{X}_{train}-{X}_{min}}{{X}_{max}-{X}_{min}}$$

3 $$\:{X}_{val}^{{\prime\:}}=\frac{{X}_{val}-{X}_{min}}{{X}_{max}-{X}_{min}}$$

where Xtrain and Xval are the original training and validation data, respectively. Min-Max Normalization is widely used in ML as it ensures that all input features are within the same range, reducing the influence of scale differences and improving model performance.

Since the input data represents spectral reflectance measurements at wavelengths ranging from 400 nm to 2000 nm in 1 nm increments, each sample is a feature vector containing 1,601 features.

### Neural architecture search

NAS has emerged as a key area within Automated Machine Learning (AutoML), which focuses on automating various stages of the ML pipeline, including data preprocessing, feature engineering, hyperparameter tuning, and model selection^33^. The ability to handle smaller datasets while optimizing model accuracy is one of the major advantages of AutoML. Additionally, this approach enables individuals with ML expertise to develop highly efficient models^34^. NAS shares significant similarities with Hyperparameter Optimization (HPO)^35^, which involves systematically optimizing a model’s hyperparameters to enhance performance. Some researchers consider NAS a subset of HPO^36^, since it specifically targets architectural hyperparameters, which form a subset of the broader set of model hyperparameters. Despite this relationship, the methodologies employed for NAS and HPO often differ significantly.

NAS is an AutoML technique designed to select the optimal neural network architecture for a given dataset to enhance model performance. The dataset utilized in the proposed work consists of a smaller number of instances; hence, the multilayer neural network is suitable for this. In the proposed work, the NAS method is categorized into the following components:

- **Search Space** – Defines the range of possible multilayer neural network architectures that can be constructed for the regression task.
- **Search Strategy** – Determines how the optimal architecture is found, including the objective function and the search methodology. In the proposed work, MAE is used as the objective function.
- **Performance Estimation Strategy** – Specifies how the performance of the generated neural network models is assessed after their construction. The performance of the neural network is evaluated in terms of MAE and R^2^.

**Fig. 2:**
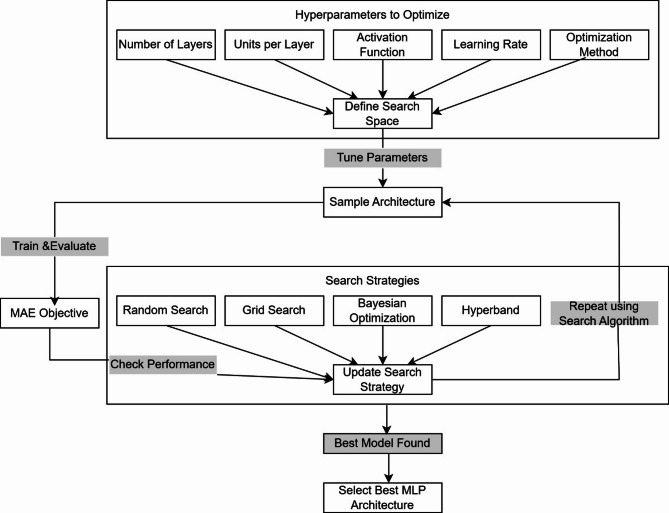
The proposed regression neural network using NAS (RegNN-NAS) architecture method.

Figure 2 depicts the steps involved in the proposed RegNN-NAS to construct the optimal neural network regressor to predict the quantity of the WW parameters. The proposed NAS constructs the neural network regressor by defining the search space, which includes the number of hidden layers, the number of neurons in each hidden layer, the activation function in each hidden layer, the optimization method, and the learning rate. All these hyperparameters define the search space.

This work utilized the four search algorithms for search strategy namely Random search, Grid Search, Bayesian optimization and Hyperband.

- Radom Search NAS Algorithm.

Radom Search algorithm is one of the simplest possible algorithms for NAS. In this case architecture are designed randomly from the defined search space and trained. In all possible architectures, the architecture with best accuracy or minimum MAE are considered.

Despite its simplicity, multiple studies^37,38^ have demonstrated that random search performs surprisingly well, particularly in highly engineered search spaces where strong architectures are prevalent. Random search algorithm design architectures withing the top 100/k% of the search space, where k represents the number of evaluations. Random search is one of recommended search strategy in evaluating NAS algorithms^39^. Algorithm 1 depicts the algorithm of random search NAS employed in proposed work.

**Figure:**
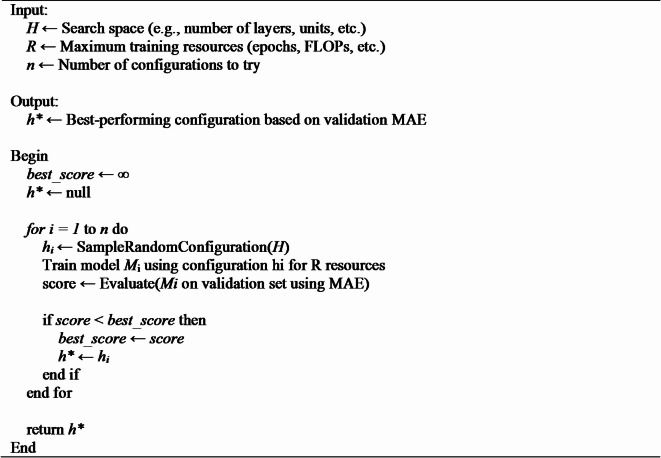
**Algorithm 1** Random Search NAS Algorithm.

b. Grid Search Algorithm for NAS.

Grid Search is the traditional approach for optimization of hyperparameters^40^. Grid search replaces the random selection of hyperparameters by exhaustively searching all possible combinations. This approach is computationally expensive for larger spaces which is best suited for small search spaces.

c. Bayesian Optimization Search for NAS.

Bayesian Optimization is an effective search technique for optimizing high-complexity black box functions^33^. This is an iterative method that designs the architecture which maximizes the acquisition function. The designed architecture undergoes the training process, and the surrogate model is updated with latest results, preparing for the next iteration. The functionality of this search algorithm consists of two factors: (i) Developing a probabilistic model – This model acts as a surrogate for the unknown objective function, using past observations to make predictions. (ii) Using an acquisition function – This helps in deciding the next points to explore by balancing exploration (trying new areas) and exploitation (focusing on promising regions)^33,41^. Algorithm 2 depicts the algorithm used for Bayesian optimization NAS.

**Figure:**
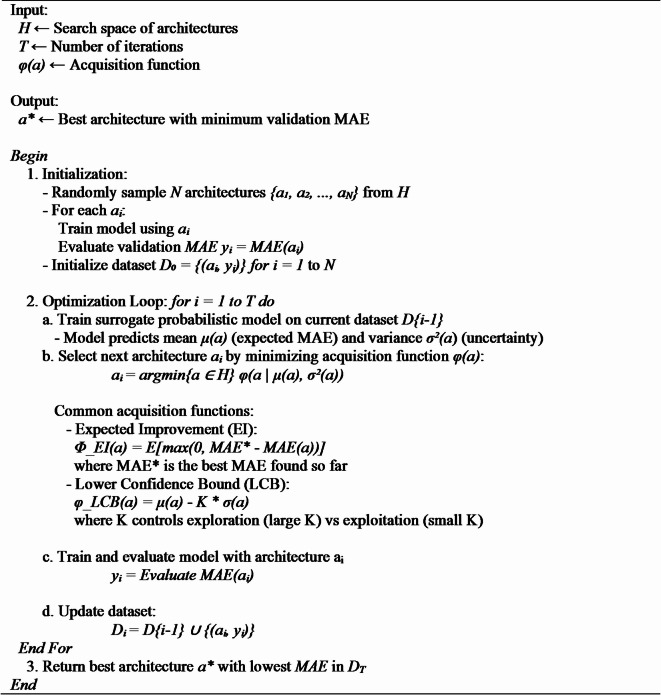
**Algorithm 2** Bayesian Optimization NAS Algorithm.

Bayesian optimization offers the advantages of low computational cost and reduced search time compared to other search strategies^41^. Bayesian Optimization is employed to efficiently explore the neural architecture or hyperparameter space. A Gaussian Process was used as the surrogate model, and the selection of the next candidate point was guided by an acquisition function. In this study, we utilized two widely adopted acquisition strategies: Lower Confidence Bound (LCB) and Expected Improvement (EI). LCB balances exploration and exploitation by prioritizing regions with either low predicted values or high uncertainty, thus promoting informed exploration of the search space. In contrast, EI selects points that are likely to yield the most improvement over the current best result, effectively trading off between high predicted performance and model uncertainty. These acquisition strategies help guide the search process intelligently, allowing the model to converge toward optimal configurations with fewer evaluations compared to exhaustive methods^42^.

d. Hyperband search.

The Hyperband algorithm is an advanced hyperparameter optimization technique designed to improve efficiency in tuning ML models^43^. It builds upon Successive Halving (SH) and is particularly useful when evaluating configurations is computationally expensive. Hyperband dynamically allocates resources to different configurations and efficiently eliminates underperforming ones. Algorithm 3 depicts the steps involved in Hyperband search algorithm^43,44^.

**Figure:**
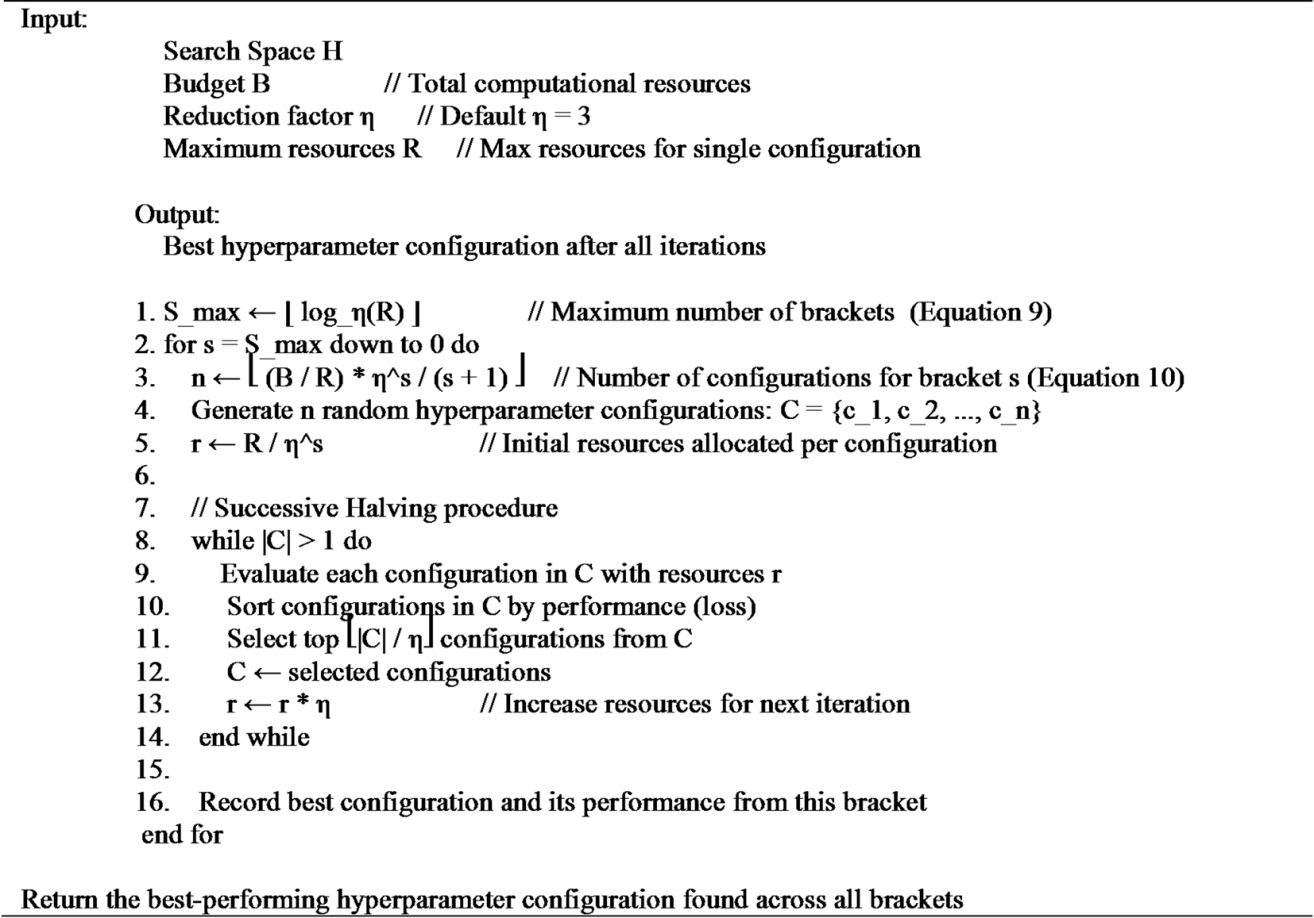
**Algorithm 3** Hyperband search NAS algorithm.

The proposed model analysed the performance of the best neural network architecture obtained by random search, grid search, Bayesian optimization and hyperband search algorithms. Table 2 depicts the comparison of these search algorithms.

**Table 2:** Comparison of NAS search algorithms.

Criteria	grid search	Random search	Bayesian optimization	Hyperband
Search type	Exhaustive	Random Sampling	Model-based Probabilistic Search	Bandit-based Resource Allocation
Learning from past trials	No	No	Yes	No
Scalability to high dimensions	Poor	Good	Moderate (can be expensive with high dimensions)	Good
Resource efficiency	Poor	Moderate	High	High
Training budget usage	Inefficient	Efficient	Very efficient	Very efficient
Computational cost	High	Medium	Medium (depends on surrogate model)	Low (prunes bad configs early)
Parallelizability	Yes	Yes	Limited (due to sequential nature of modeling)	Yes
Implementation complexity	Simple	Simple	Moderate-to-High (model + acquisition function)	Moderate
Convergence speed	Slow	Medium	Fast (when model is accurate)	Fast (especially for deep learning models)
Suitability for NAS	Low	Medium	High	High
Key limitation	Combinatorial explosion	Can miss good configs randomly	Surrogate model may not scale or may mispredict	Requires well-defined budget/resource allocation
Example toolkits	scikit-learn, KerasTuner	KerasTuner, scikit-learn	Optuna, SMAC, Hyperopt, Spearmint	KerasTuner, Ray Tune,

To evaluate whether different hyperparameter tuning strategies produce significantly different predictive performances, we applied both parametric and non-parametric statistical tests. One-way ANOVA and Kruskal-Wallis tests were conducted per individual target variable to assess differences among tuners. Additionally, the Friedman test was applied across all target variables to examine overall tuner performance differences.

### Explainable artificial intelligence (XAI) for interpretation of neural network decision

Explainable AI(XAI) techniques are crucial in this aspect by providing an intuitive understanding of AI decisions^45^. XAI improves the dependability and trustworthiness of AI-generated outputs by offering clear understanding of how a model reaches its conclusions. XAI helps identify what aspects of an input are raising or lowering a model’s prediction. After these facts, Users can make better decisions by interpreting the AI outputs^46^. In the proposed work, local interpretable model- agnostic explanations (LIME) approach is applied to resolve the issue of interpretability of the ML models. It focuses on two major things: the model’s confidence and the reliability of individual predictions. By offering explanations at a local level, LIME helps bridge the gap between AI decision-making and human interpretation^47^.

**Fig. 3:**
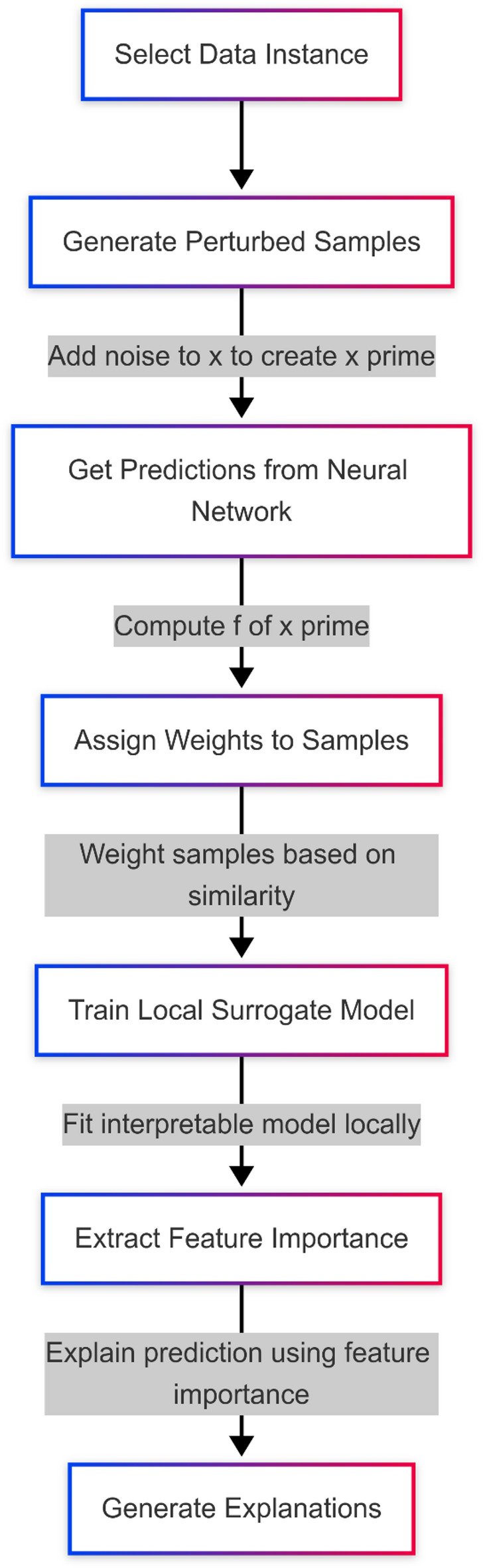
Flow of LIME XAI approach.

The flowchart described in Fig. 3 depicts the representation of the LIME algorithm, the LIME algorithm is used to explain the prediction of black-box ML models like neural networks by approximating the prediction locally by an interpretable model. It starts by choosing a specific data instance *x* to explain. To visualize the decision boundary of the model around x, perturbed samples x′ are generated by adding random noise — often drawn from a normal distribution according to Eq. 4.

4 $$\:{x}^{{\prime\:}}=x+\in\:$$

where ε ∼N(0,σ2). It then feeds these perturbed samples into the neural network to obtain the predictions y’=f(x’), where f is the model of the black box.

Then the perturbed samples are weighted according to their closeness to the original instance with a kernel function, usually an exponential kernel function, as indicated in Eq. 5.

5 $$\:{\pi\:}_{x}\left({x}^{{\prime\:}}\right)=\text{e}\text{x}\text{p}(-\frac{{d\left(x,{x}^{{\prime\:}}\right)}^{2}}{{\sigma\:}^{2}})$$

where $\:d\left(x,{x}^{{\prime\:}}\right)$ is a chosen distance metric. The next step is to fit a simpler, local Neural Network Surrogate Model g(x) on the weighted dataset, which is intended to imitate the behaviour of the black-box neural network but strictly within the neighbourhood of x. To help interpret this concept, the surrogate model^48^can be seen as a simplified proxy that approximates the original complex model’s behaviour — but only in a very narrow region around the input instance. It helps identify which features are most influential in that specific case. This local perspective is what makes LIME valuable for interpretability. By focusing only on the ‘local neighbourhood’ of a prediction, it avoids the complexity of explaining the entire model, similar to using a zoomed-in map to understand a single area instead of the whole terrain.

Instead of fitting a simple linear regression model, the surrogate model here is a small, interpretable neural network, comprising only a few layers and having limited complexity, to ensure it manages to learn meaningful local patterns while remaining interpretable. The training losses are minimized on this weighted mean squared errors. The goal is to minimize a weighted loss function defined by Eq. 6.

6 $$\:\mathcal{L}\left(f,\:g,\:{\pi\:}_{x}\right)=\:\sum\:_{{x}^{{\prime\:}}\:\in\:{X}^{{\prime\:}}}{\pi\:}_{x}\left({x}^{{\prime\:}}\right){\left(f{(x}^{{\prime\:}}\right)-g{(x}^{{\prime\:}}\left)\right)}^{2}$$

This makes sure that the predictions of the surrogate model follow closely the predictions of the original black-box neural network but only in the local region of interest. We consider gradient-based methods of feature importance extraction after training the surrogate model. The importance of feature x_i​_ is computed as in Eq. 7, where g(x) is the output of a neural network function^40^.

7 $$\:Feature\:\:Importance\:\left({x}_{i}\right)=\left|\frac{\partial\:g\left(x\right)}{\partial\:{x}_{i}}\right|$$

These importance values explain which features contributed the most to the prediction at *x*. Finally, these explanations are presented to the user in an interpretable form, helping them understand the behaviour of the neural network regressor locally around x. This method makes it possible to explain complex neural network regression models while preserving their predictive power.

It is important to distinguish between global and local interpretability. Global interpretability refers to understanding the model’s behavior across the entire input space, while local interpretability, as used in LIME, focuses on explaining individual predictions by approximating the model’s behavior near a specific instance. This localized explanation is particularly valuable in complex, high-dimensional models like neural networks, where global understanding is often infeasible. In this study, LIME is employed because the focus is to understand the model predictions for individual events in the dataset, such as sudden anomalies in wastewater parameters, rather than interpreting the model’s full behaviour globally.

### Statistical analysis

To compare the performance of multiple hyperparameter tuning strategies across different target variables, we employed non-parametric statistical tests recommended for machine learning model comparisons^49^. When evaluating K models over N datasets, simple pairwise tests such as the Wilcoxon signed-rank test may inflate the Type I error rate due to multiple comparisons. Therefore, we adopted the Friedman test, which assesses whether the differences in average ranks of models are statistically significant across datasets. For each tuner j, the models were ranked based on their evaluation metric (R^2^), producing ranks $\:{r}_{j,k}$ ​ for model k. The average rank for each model is computed as shown in the Eq. 8.

8 $$\:{\stackrel{-}{r}}_{k}=\frac{1}{N}\sum\:_{j=1}^{N}{r}_{j,k}$$

The Friedman test statistic is then calculated by Eq. 9.

9 $$\:{\chi\:}_{F}^{2}=\frac{12N}{K(K+1)}\sum\:_{k=1}^{K}{\left({\stackrel{-}{r}}_{k}-\frac{k+1}{2}\right)}^{2}$$

Under the null hypothesis that all models perform equivalently, ​ $\:{\chi\:}_{F}^{2}\:$follows a chi-square distribution with K − 1 degrees of freedom.

To reduce the conservativeness of this test, the Iman-Davenport correction transforms the Friedman statistic into an F-distributed statistic as shown in Eq. 10.

10 $$\:{F}_{F}=\:\frac{(N-1){\chi\:}_{F}^{2}}{N\left(K-1\right)-{\chi\:}_{F}^{2}}$$

which follows an F-distribution with K − 1 and (K − 1)(*N* − 1) degrees of freedom.

Upon finding significant differences via Friedman’s test, post-hoc pairwise comparisons were conducted using the Wilcoxon signed-rank test. To control for the increased risk of Type I error due to multiple testing, Bonferroni correction was applied to the significance level.

This combination of tests ensures robust and reliable comparison of the multiple tuning strategies across various targets.

## Methodological limitations

While the proposed approach demonstrates promising results in predicting multiple wastewater parameters, several methodological limitations and assumptions must be acknowledged:

1. Limited Dataset Size:

The dataset used consists of small number of samples, which may not fully capture all operational variances in real-world wastewater treatment plants. As a result, the model’s generalizability to unseen or extreme scenarios may be limited.

2. No Cross-Validation Due to Small Dataset:

Given the limited data size, we adopted a hold-out validation approach instead of cross-validation to avoid overfitting and ensure fair comparison across neural architecture search strategies. This decision prioritizes computational feasibility but may affect robustness.

3. Assumption of Stationarity:

The model assumes that the statistical properties of the data remain constant over time. However, wastewater characteristics can vary seasonally or due to operational changes. This could affect prediction accuracy in dynamic environments.

## Results and analysis

### Experimental conditions

This work focuses on predicting wastewater characteristics using a dataset proposed by the authors of^14^. The dataset includes various influent wastewater parameters, and the study aims to predict key characterization metrics, including BOD, COD, NH_3_-N, TDS, TA, and TH concentrations.

To conduct the experiments, the dataset was split into training and testing sets in a 90:10 ratio. The model was trained on 90% of the data, while the remaining 10% was used for testing to evaluate performance.

To optimize the neural network architecture, AutoML-based NAS was employed using four different search strategies: Random Search, Grid Search, Bayesian Optimization, and Hyperband. The search strategies were used to tune the model’s hyperparameters efficiently, ensuring an optimized neural network for both single-target and multi-target prediction.

All models were implemented using TensorFlow and Keras libraries in Python, and training was carried out on a Google Colaboratory (Colab), a cloud-based Jupyter notebook service provided by Google. The runtime environment was configured with: 12th Gen Intel(R) Core(TM) i5-1235, 8GB RAM.

The hyperparameters for the neural network were carefully selected based on prior observations and experimentation. The selected ranges are depicted in Table 3. The neural network hyperparameter space was designed based on a comprehensive review of various neural network topologies used in wastewater prediction studies, as detailed in^50^.

**Table 3:** Neural network hyperparameter search space.

Neural network hyperparameters	Minimum	Maximum	Steps/options
Number of hidden layers	1	5	1 (integer step)
Number of neurons per hidden layer	30	1000	2 (integer step)
Learning rate	1e^− 5^	1e^− 2^	Log sampling
Activation function in hidden layer	ReLu, ELU, LeakyReLu, Selu		Chosen based on best performance
Optimization algorithm	Adam and Adagrad		Selected for adaptive learning rates

The hyperparameter selections used in this study was selected to maximize the performance of the neural network. The number of hidden layers will search between 1 and 5, in order to have flexibility in the networks depth, thus having different levels of model complexity. In the same vein, number of neurons per layer were set between 30 and 1000, and step size was set at 2, allowing for both wide exploration as well as narrowing down on fine-tuned architecture. The learning rate that heavily affects model convergence was selected to vary from 1e-5 to 1e-2 using a log sampling design to allow for an effective optimization procedure. Four activation functions ( ReLU, ELU, LeakyReLU, and SeLU ) were examined for their ability to improve gradient flow and thus ultimately achieve better network stability. Additionally, two optimization algorithms, Adam and Adagrad, were tested, as both dynamically adjust learning rates to improve convergence and overall model performance.

Four different NAS search algorithms were used to find the best-performing model architecture. The parameters for each are listed Table 4.

**Table 4:** Search algorithm parameters.

Search algorithm	Max trials	Executions per trial	Other parameters
Random search	10	5	–
Grid search	10	5	–
Bayesian optimization	10	5	–
Hyperband	10	5	Max Epochs = 50, Factor = 3

The study employed four different search strategies to optimize the neural network architecture efficiently. Random Search randomly samples hyperparameters within the defined ranges for 10 trials, with 5 executions per trial to ensure stability and reduce variance in results. Grid Search systematically explores all possible hyperparameter combinations within the given space, ensuring comprehensive coverage but requiring higher computational resources. Bayesian Optimization leverages probabilistic models to prioritize hyperparameter selections that are more likely to enhance performance, making it a more efficient alternative to random search. Hyperband dynamically allocates computational resources by focusing on the most promising model configurations. It employs a maximum of 50 epochs with a factor of 3, allowing early termination of underperforming models, thereby optimizing computational efficiency while maintaining high predictive performance.

### Neural network for prediction of single target

This study focused on predicting both single and multiple targets, with an optimized neural network architecture designed for each case. Tables 5, 6, 7 and 8 presents the architectural parameters of neural networks obtained through NAS using random search, grid search, Bayesian optimization, and Hyperband search algorithms, respectively, for single-target predictions.

**Table 5:** Optimal neural network parameters for single-target prediction using random search algorithm.

Search algorithm: random search	
Target	Neural network architecture
BOD	{‘num_layers’: 2, ‘units_0’: 740, ‘learning_rate’: 0.0004489080930912622, ‘units_1’: 798, activation function: Relu }
COD	{‘num_layers’: 5, ‘units_0’: 946, ‘learning_rate’: 0.00013877861489844856, ‘units_1’: 746, ‘units_2’: 832, ‘units_3’: 802, ‘units_4’: 916, activation function: Relu }
NH_3_-N	{‘num_layers’: 1, ‘units_0’: 560, ‘learning_rate’: 0.0006820026103064649, ‘‘ activation function: Relu’}
TDS	
TA	
TH	{‘num_layers’: 2, ‘units_0’: 730, ‘learning_rate’: 6.0681102045712135e-05, ‘units_1’: 104, ‘ activation function: Relu’}

**Table 6:** Optimal neural network parameters for single-target prediction using grid search algorithm.

Search algorithm: grid search	
Target	Neural network architecture
BOD	{‘num_layers’: 1, ‘units_0’: 10, ‘learning_rate’: 0.0007943282347242817, ‘ activation function: Relu’}
COD	{‘num_layers’: 1, ‘units_0’: 30, ‘learning_rate’: 0.0007943282347242817, ‘ activation function: Relu’}
NH_3_-N	
TDS	
TA	
TH	{‘num_layers’: 1, ‘units_0’: 30, ‘learning_rate’: 0.00012589254117941674, ‘ activation function: Relu’}

**Table 7:** Optimal neural network parameters for single-target prediction using bayesian search algorithm.

Search algorithm: Bayesian optimization	
Target	Neural network architecture
BOD	{‘num_layers’: 3, ‘units_0’: 202, ‘learning_rate’: 0.0009673431123937811, ‘units_1’: 882, ‘units_2’: 388}
COD	{‘num_layers’: 4, ‘units_0’: 680, ‘learning_rate’: 0.0008075139307516213, ‘units_1’: 534, ‘units_2’: 432, ‘units_3’: 220, ‘ activation function: Relu’}
NH_3_-N	
TDS	
TA	
TH	{‘num_layers’: 1, ‘units_0’: 220, ‘learning_rate’: 0.0001060864701413733, ‘ activation function: Relu’}

**Table 8:** Optimal neural network parameters for single-target prediction using hyperband search algorithm.

Search algorithm: Hyperband	
Target	Neural network architecture
BOD	{‘num_layers’: 3, ‘units_0’: 356, ‘learning_rate’: 0.0007704712206420708, ‘units_1’: 340, ‘units_2’: 608, ‘ activation function: Relu’, ‘tuner/epochs’: 50, ‘tuner/initial_epoch’: 17, ‘tuner/bracket’: 3, ‘tuner/round’: 3, ‘tuner/trial_id’: ‘0046’}
COD	{‘num_layers’: 3, ‘units_0’: 194, ‘learning_rate’: 0.0009133974940373165, ‘units_1’: 794, ‘units_2’: 988, ‘activation function: Relu’, ‘tuner/epochs’: 50, ‘tuner/initial_epoch’: 17, ‘tuner/bracket’: 2, ‘tuner/round’: 2, ‘tuner/trial_id’: ‘0067’}
NH_3_-N	
TDS	
TA	
TH	{‘num_layers’: 4, ‘units_0’: 336, ‘learning_rate’: 0.0001803296783831608, ‘units_1’: 102, ‘units_2’: 178, ‘units_3’: 50, ‘activation function: Relu’, ‘tuner/epochs’: 50, ‘tuner/initial_epoch’: 17, ‘tuner/bracket’: 2, ‘tuner/round’: 2, ‘tuner/trial_id’: ‘0069’}

Using the optimal parameters described in Tables 5, 6, 7 and 8, the neural network predicted the values of single targets. The performance of the optimal neural network obtained by each of the search algorithm is measured using mean absolute error and R^2^.

**Fig. 4:**
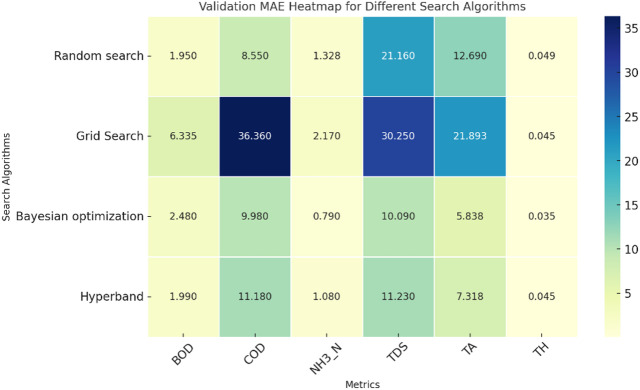
Validation MAE of single target value prediction using various search algorithms.

**Fig. 5:**
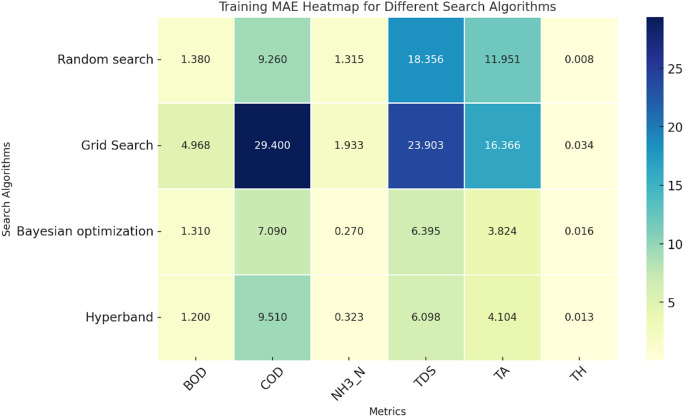
Training MAE of single target value prediction using various search algorithms.

The comparison of the validation and training MAE heatmaps from Figs. 4 and 5 reveals key insights into the performance of different hyperparameter search algorithms. Grid Search consistently shows the highest MAE values across both training and validation, particularly for COD, TDS, and TA, indicating poor generalization and a tendency to overfit. Bayesian Optimization, on the other hand, demonstrates the lowest MAE values across most metrics, making it the most effective method for hyperparameter tuning. Random Search and Hyperband exhibit relatively low errors, but Bayesian Optimization consistently outperforms them, especially in NH_3_-N, TDS, and TA. A notable observation is the significant drop in performance from training to validation for Grid Search, particularly in COD (29.4 to 36.36) and TA (16.366 to 21.893), which further indicates overfitting. Bayesian Optimization, however, maintains stable error values, suggesting better generalization across datasets. Hyperband and Random Search show moderate generalization with slight increases in validation error compared to training, but their performance remains reasonable.

Overall, Bayesian Optimization emerges as the most effective search algorithm due to its ability to maintain low errors across both datasets, ensuring better generalization. Hyperband follows as a promising alternative, while Grid Search proves to be the least effective, given its high errors and signs of overfitting. To improve model performance, Bayesian Optimization should be prioritized, and Grid Search should be avoided for this dataset. Additionally, NH_3_-N and COD require careful monitoring, as they exhibit significant variations across different methods.

**Fig. 6:**
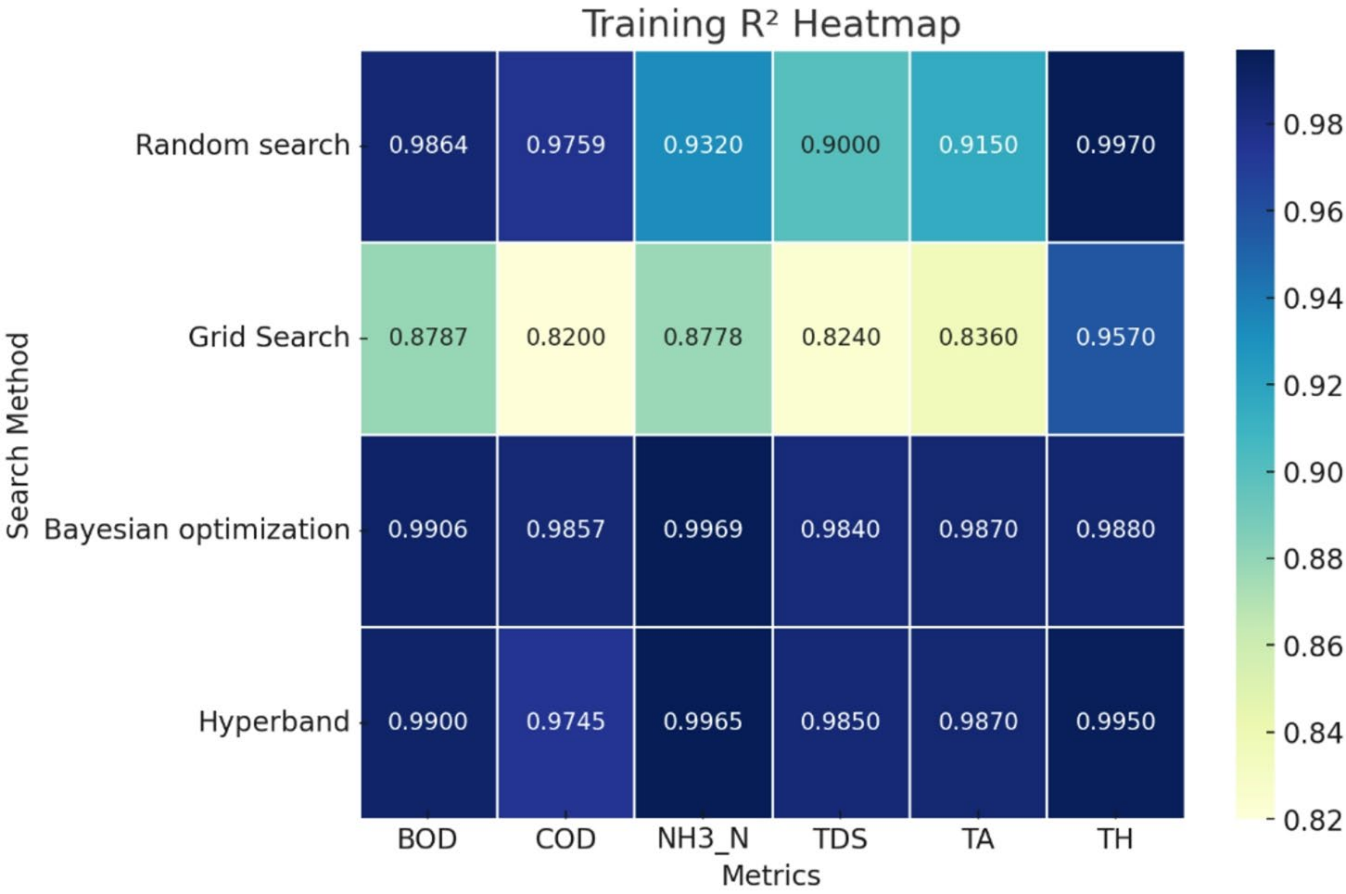
Training R^2^ of single target value prediction using various search algorithms.

**Fig. 7:**
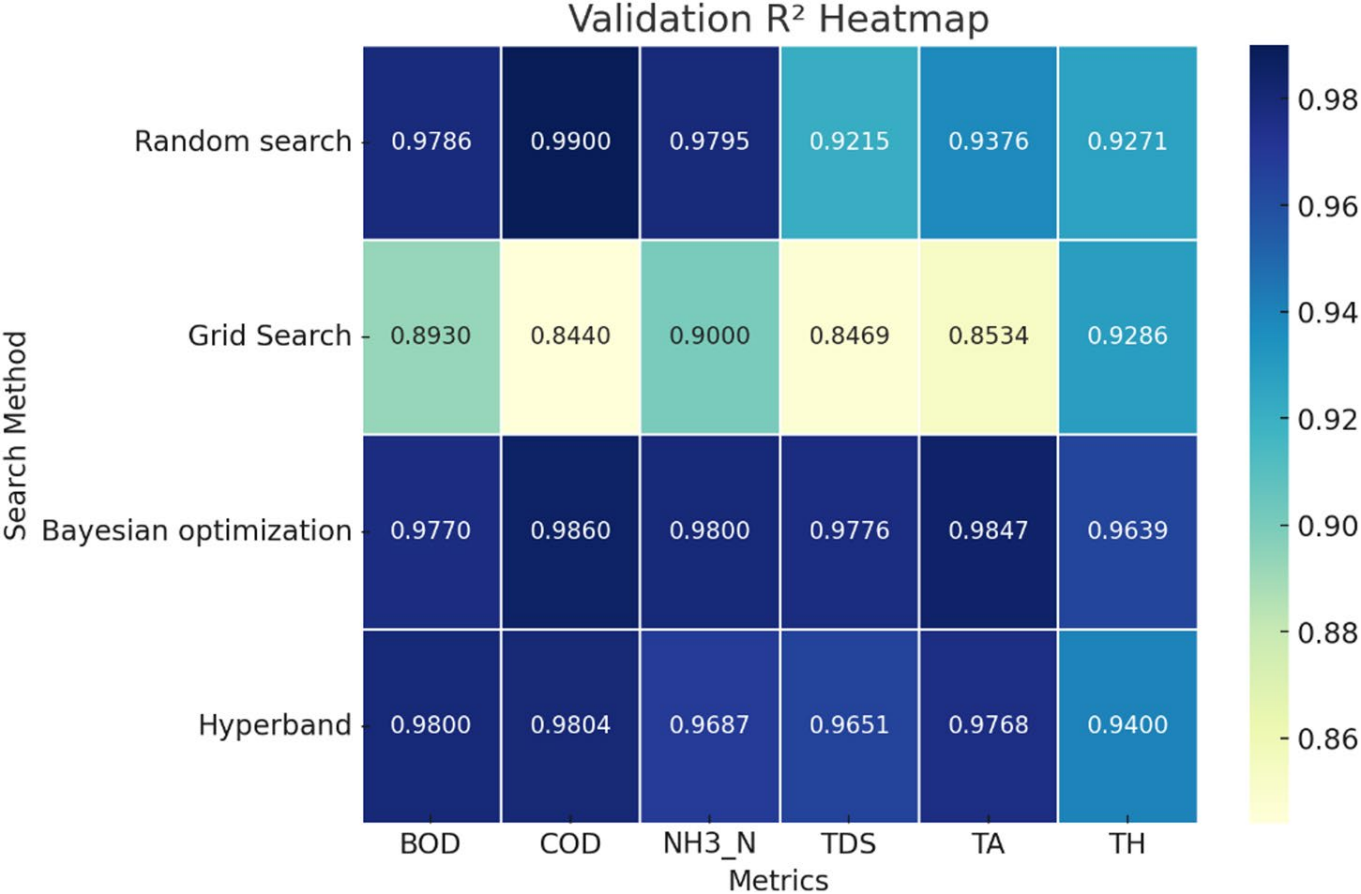
Validation R^2^ of single target value prediction using various search algorithms.

For single-target prediction, the analysis of training and validation R^2^ values across different hyperparameter optimization methods from Figs. 6 and 7 reveals distinct trends in model performance. Random Search demonstrates consistently high R^2^ values in both training and validation, indicating strong predictive capabilities, but slight drops in NH_3__N and TDS suggest minor overfitting. Grid Search, however, exhibits the weakest performance, with significantly lower R^2^ values in both training and validation, particularly for COD and NH_3__N, making it the least effective approach. Bayesian Optimization achieves the highest R^2^ values across both training and validation, ensuring not only excellent model fit but also strong generalization, making it an optimal choice. Hyperband follows closely, performing comparably to Bayesian Optimization, though slight validation dips in NH_3_-N and TDS suggest minor overfitting.

When considering both MAE and R^2^ together, Bayesian Optimization emerges as the best-performing method. It achieves the lowest MAE across most parameters, indicating minimal prediction errors, while maintaining consistently high R^2^ values, which signifies strong model accuracy and generalization. Hyperband performs well but has slightly higher MAE values than Bayesian Optimization, making it the second-best option. Random Search, although achieving decent R^2^ values, has relatively higher MAE, particularly in some parameters, suggesting that its predictions are not as precise. Grid Search performs the worst overall, with the highest MAE and the lowest R^2^ values, signifying poor predictive accuracy and underfitting. Therefore, Bayesian Optimization is the best method, as it balances low MAE with high R^2^, ensuring both precision and generalization in single-target regression tasks.

### Multi-target prediction using neural network regression

Neural networks can be designed for multi-target regression, where a single model predicts multiple output values simultaneously. In the context of wastewater parameter estimation, this means that a single NN model is trained to predict multiple WW parameters (e.g., BOD, COD, NH_3__N, TDS, TA, and TH) instead of training separate models for each parameter.

While multi-target regression offers computational efficiency and reduced training time, it often comes with trade-offs in prediction accuracy. In this study, NN models were trained with 2 to 6 output parameters, and their optimal architectures were determined using NAS by analyzing R^2^ and MAE. By leveraging NAS, the optimal architecture for each case was efficiently identified, ensuring that the model was neither over-parameterized nor underperforming. Table 9 depicts the optimal architectural parameters obtained by Random Search, Grid Search, Bayesian Optimization, and Hyperband, respectively, for multiple target prediction.

**Table 9:** Optimal neural network parameters for multiple-targets prediction using various search algorithm.

Search algorithm	Best hyperparameters/targets	Number of layers	Units_1	Units_2	Units_3	Units_4	Units_5	Learning rate
Random Search	BOD, COD	2	746	802	–	–	–	0.0030077
	BOD, COD, NH_3_-N							
	BOD, COD, NH_3_-N, TDS							
	BOD, COD, NH_3_-N, TDS, TA							
	BOD, COD, NH_3_-N, TDS, TA, TH	5	946	746	832	802	916	0.0005169
Grid Search	BOD, COD	1	30		–	–	–	0.003548134
	BOD, COD, NH_3_-N							
	BOD, COD, NH_3_-N, TDS							
	BOD, COD, NH_3_-N, TDS, TA							
	BOD, COD, NH_3_-N, TDS, TA, TH							
Bayesian optimization search	BOD, COD	2	48	682	–	–	–	0.001538364
	BOD, COD, NH_3_-N							
	BOD, COD, NH_3_-N, TDS							
	BOD, COD, NH_3_-N, TDS, TA							
	BOD, COD, NH_3_-N, TDS, TA, TH	3	396	986	228			0.00089288
Hyperband search	BOD, COD	3	936	194	370	–	–	0.00591
	BOD, COD, NH_3_-N							
	BOD, COD, NH_3_-N, TDS							
	BOD, COD, NH_3_-N, TDS, TA							
	BOD, COD, NH_3_-N, TDS, TA, TH	5	938	554	534	352	552	0.002988

The analysis of optimal neural network parameters for multiple-target prediction using different hyperparameter search algorithms—Random Search, Grid Search, Bayesian Optimization, and Hyperband—reveals notable variations in network architectures and learning rates across different target combinations. For the prediction of BOD and COD, the Random Search algorithm selects a two-layer architecture with 746 and 802 units, a learning rate of 0.0030077, and the ReLU activation function, whereas the Grid Search algorithm opts for a simpler one-layer network with only 30 units and a slightly higher learning rate of 0.003548134. Bayesian Optimization, in contrast, finds an optimal two-layer architecture with 48 and 682 units, using a lower learning rate of 0.001538364. Meanwhile, the Hyperband algorithm identifies a three-layer network with 936, 194, and 370 units and a relatively high learning rate of 0.00591.

For the most complex target combination (BOD, COD, NH_3_-N, TDS, TA, TH), the Random Search algorithm selects a five-layer network with varying units (946, 746, 832, 802, and 916) and a notably low learning rate of 0.0005169. Bayesian Optimization also finds a multi-layer architecture with three layers (396, 986, and 228 units) and a learning rate of 0.00089288. Hyperband, however, selects a five-layer structure with different unit distributions (938, 554, 534, 352, and 552) and a relatively higher learning rate of 0.002988. Notably, Grid Search does not provide optimal configurations for multi-target combinations beyond BOD and COD, which may suggest inefficiency in tuning hyperparameters for more complex prediction tasks. Across all algorithms, the ReLU activation function is consistently used in all hidden layers, and 1000 epochs are applied in each case, ensuring sufficient training iterations for convergence. The differences in architecture depth, number of units, and learning rates indicate that different search strategies prioritize different trade-offs between model complexity and optimization efficiency.

**Fig. 8:**
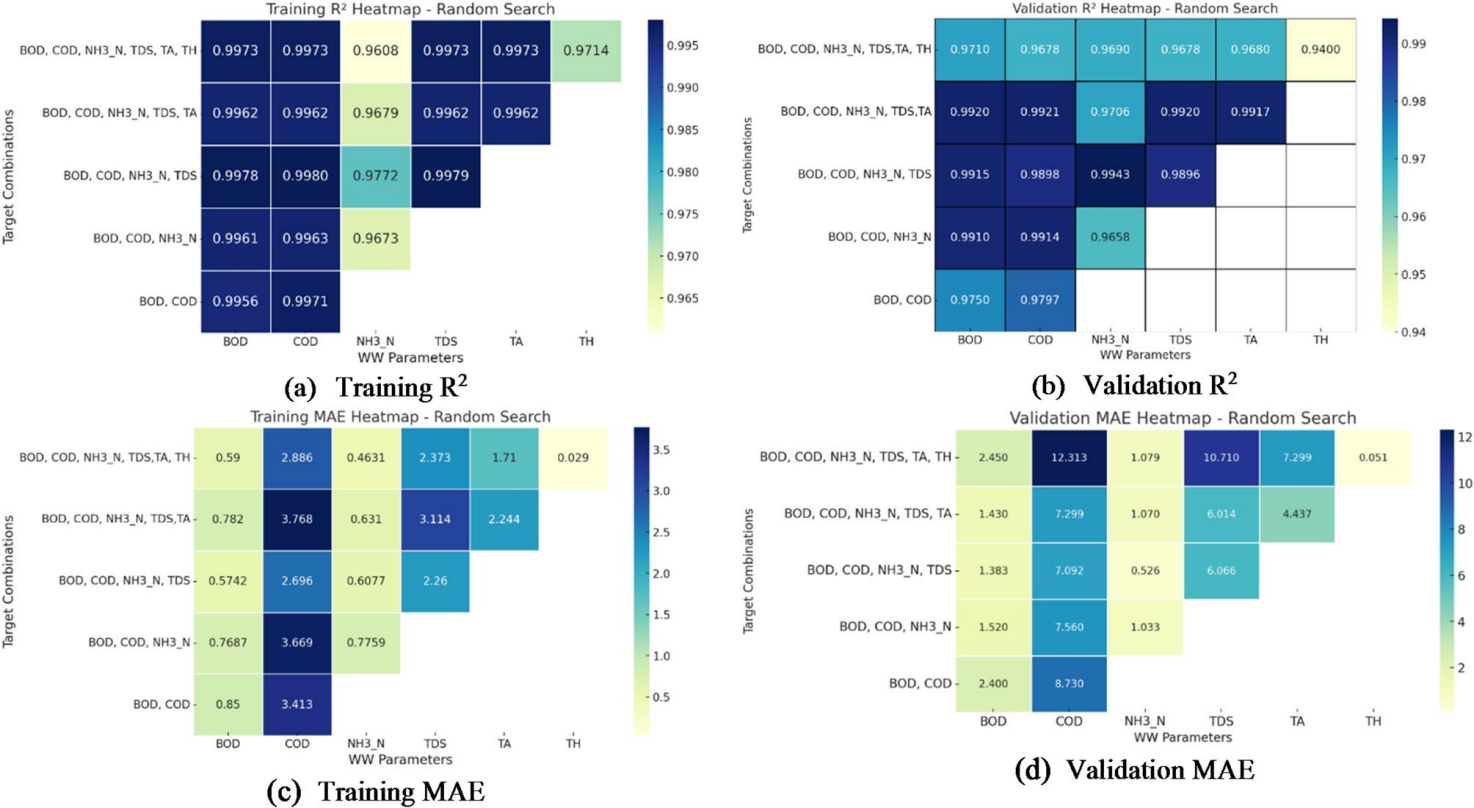
(**a**–**d**) Performance evaluation of random search: R^2^ and MAE heatmaps for training and validation.

The analysis of the training and validation heatmaps from Fig. 8a–d reveals key insights into the model’s performance using random search. The training R^2^ values indicate that the model fits the data well, with most parameters achieving values above 0.99. However, NH_3_-N and TH show slightly lower R^2^values, around 0.96–0.97, suggesting that these parameters might be more challenging to predict accurately. In the validation phase, a slight drop in R^2^is observed, particularly for NH_3_-N and TH, where values decline to around 0.94–0.97. This decrease indicates that the model may not generalize as well for these specific parameters, while BOD and COD maintain relatively strong performance across both training and validation.

The MAE heatmaps further highlight discrepancies in prediction accuracy. During training, BOD and NH_3_-N exhibit the lowest errors, indicating they are well predicted, whereas COD shows relatively higher MAE values (~ 2.6–3.7), suggesting some difficulty in precise prediction. Interestingly, TH has an extremely low training MAE (~ 0.029), indicating high predictability within the training set. However, in the validation phase, COD exhibits a significant increase in MAE (12.3), suggesting overfitting, as its training error is much lower. NH_3_-N and TDS maintain stable performance with lower validation MAE values (~ 1.0), whereas TH continues to have a very low MAE (~ 0.051), indicating consistency in prediction accuracy. The overall results suggest that while the model performs well for most parameters, NH_3_-N and TH show slightly weaker predictive capabilities, and COD experiences overfitting, leading to increased validation error.

**Fig. 9:**
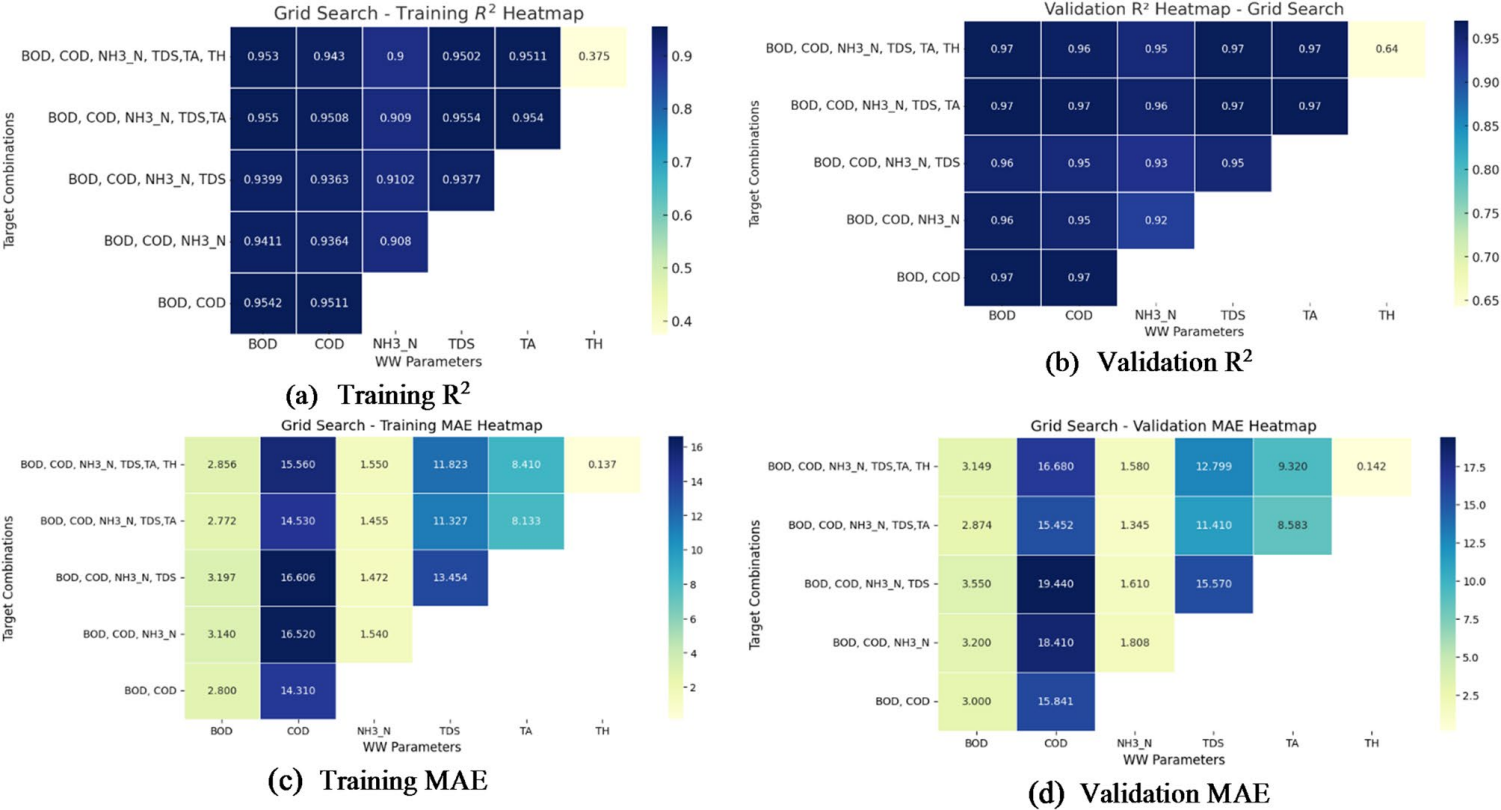
(**a**–**d**) Performance evaluation of Grid search: R^2^ and MAE heatmaps for training and validation.

The analysis of the training and validation heatmaps from Fig. 9a–d reveals key insights into the model’s performance using Grid Search. The training R^2^ heatmap shows that the model performs well for BOD and COD, with R^2^ values ranging between 0.9411 and 0.9552 when using all input parameters. However, the performance for TH is significantly lower, with an R^2^ value dropping to 0.375, indicating poor predictability. NH_3_-N, TDS, and TA exhibit moderate to high R^2^ values (0.902 to 0.9502), suggesting a relatively better fit.

The results during validation follow a similar pattern compared to the training results and indicate good generalization for all but a few of the target variables. BOD, COD, and NH_3_-N remain approximately constant with R^2^ values of about 0.96–0.97, confirming that the model is reliable for predicting these parameters. However, TH again exhibits the lowest validation R^2^, indicating that the model has not characterized the variability of this parameter efficiently. More details about prediction errors are visible in the MAE heatmaps. During training, COD has the highest error when fewer input parameters are used, since more features are needed to achieve an accurate prediction. MAE values for NH_3_-N, TDS, and TA are considered moderate, while BOD has low errors.

Next, in the validation phase, we find that the MAE for COD is relatively high, which reaffirms that this target is a difficult variable to predict even at high R^2^ values. Due to the reasonable but moderate error for NH_3_-N and TDS, and the low MAE with low R^2^ for TH, the model indicates moderate explanation of the variance. Although absolute errors are low, the absolute error for TDS is low but shows imbalanced performance due to limited explanation of the residual variance.

**Fig. 10:**
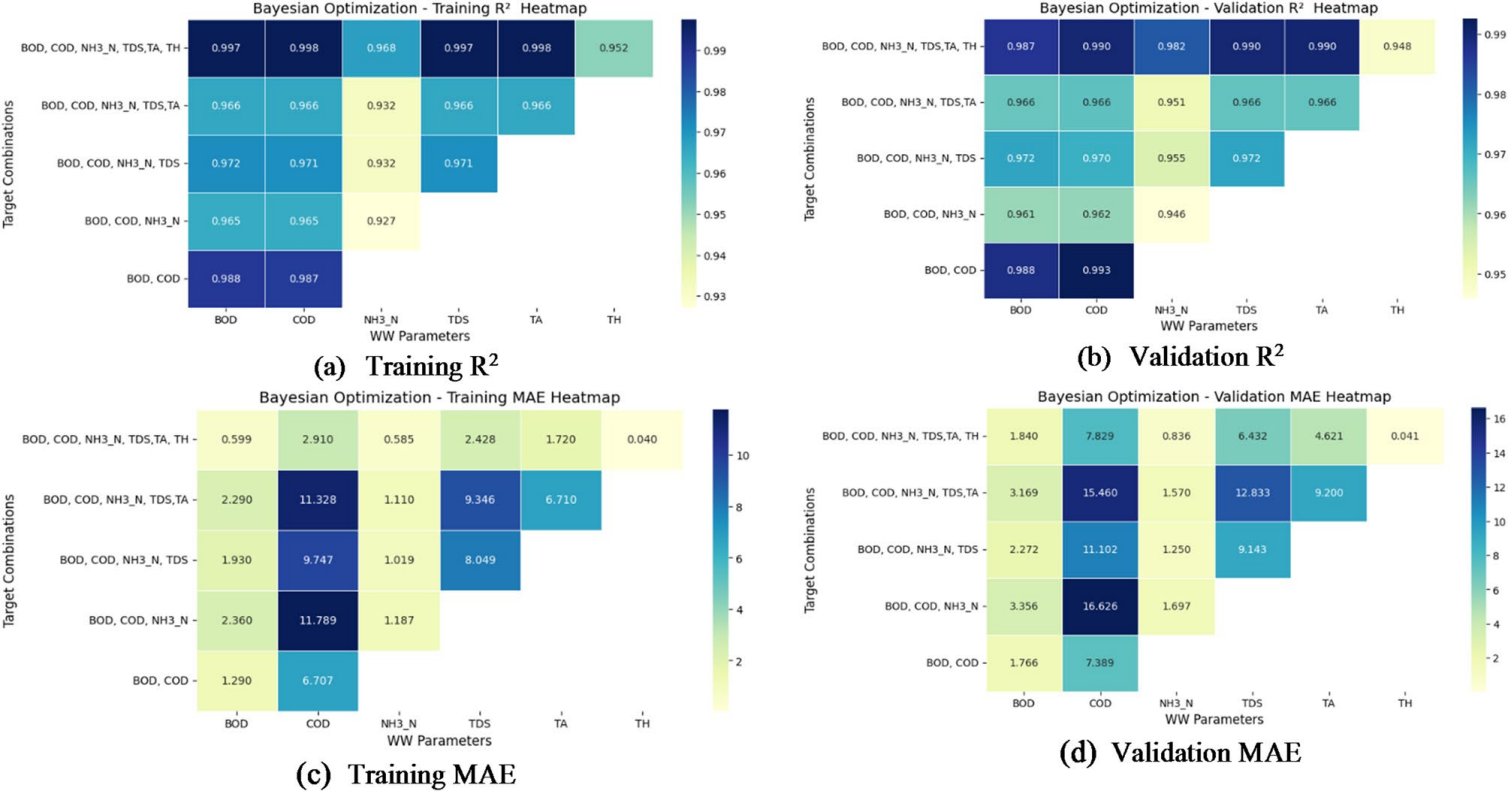
(**a**–**d**) Performance evaluation of Bayesian Optimization search: R^2^ and MAE heatmaps for training and validation.

The R^2^ and MAE heatmaps of the training and validation performance visualizations depicted in Fig. 10a–d for the prediction of multi-target regression using Bayesian Optimization. From the training R^2^ heatmap, it is evident that a high-level accuracy BOD, COD and NH_3_-N is achieved using Bayesian Optimization ( R^2^ > 0.96). The model also achieves further performance for TDS, TA and TH, showing an improved R^2^ value of 0.80 for TH in comparison to the previous methods. The R^2^ heatmap for validation confirms this consistency in performance maintaining high values for most targets, except for TH with an R^2^ value of 0.718 indicating some difficulty in capturing its variability.

The MAE heatmaps provide further insights into prediction errors. The training MAE heatmap shows low errors for BOD and COD but relatively higher errors for NH_3_-N, TDS, and TA. TH has the lowest training MAE (0.075), which aligns with its lower R^2^, suggesting that while the model minimizes absolute errors, it does not fully explain the variance in TH. The validation MAE heatmap follows a similar trend, with NH_3_-N and COD exhibiting higher errors, while BOD and TH have lower MAE values. Despite some variations, Bayesian Optimization demonstrates robust predictive performance, particularly for BOD, COD, and NH_3_-N, while showing improvements for TH compared to previous approaches.

**Fig. 11:**
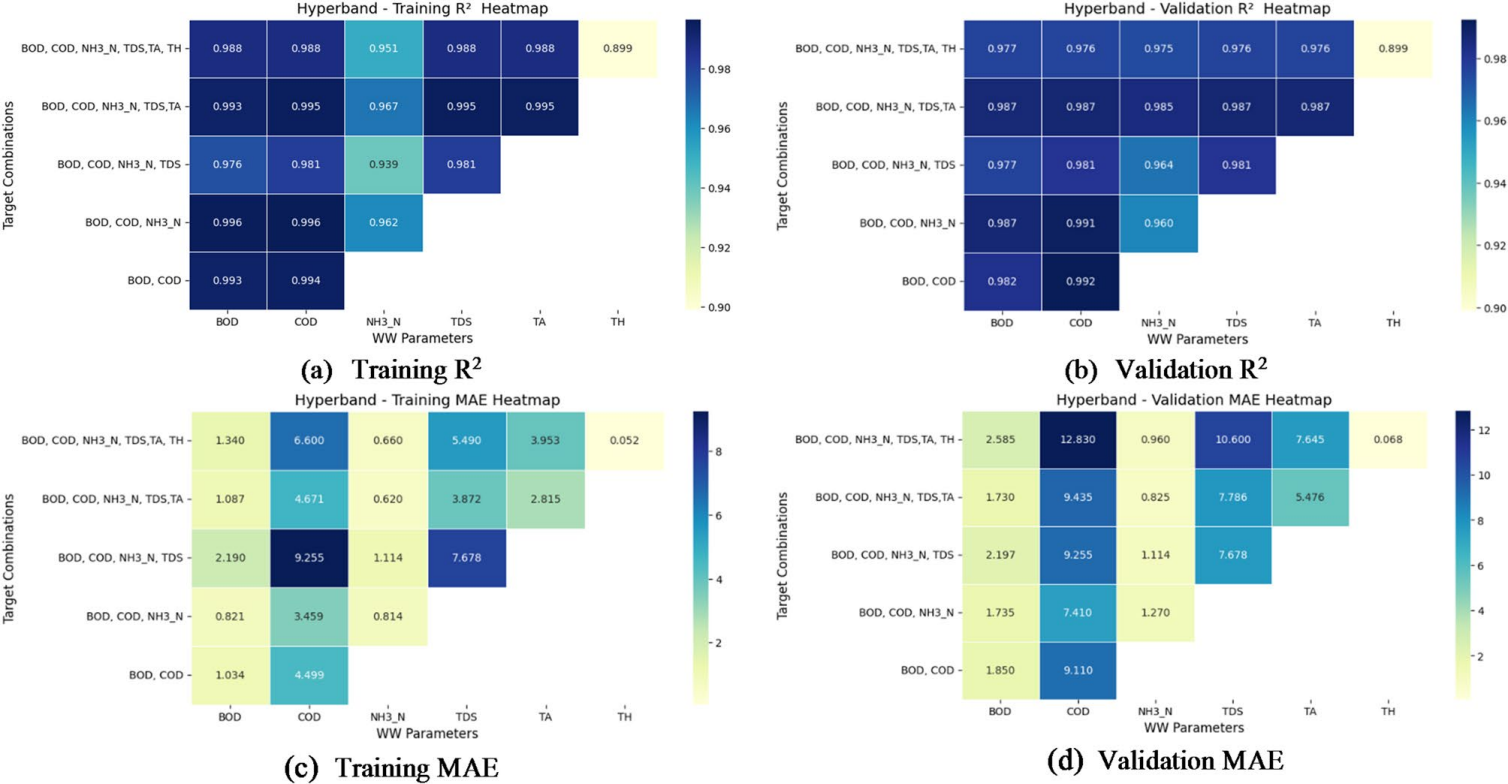
(**a**–**d**) Performance evaluation of Hyperband search: R^2^ and MAE heatmaps for training and validation.

The performance evaluation of Hyperband for multi-target regression prediction is analysed using R^2^ and MAE heatmaps for training and validation, which are shown in Fig. 11a–d. The training R^2^ heatmap demonstrates consistently high values across all target combinations, particularly for BOD, COD, and NH_3_-N, with R^2^ values above 0.95. The model also performs well for TDS, TA, and TH, with TH achieving an R^2^ of 0.899, indicating reasonable predictive capability. The validation R^2^ heatmap follows a similar trend, showing robust generalization with R^2^ values above 0.96 for most parameters, except for TH, which retains an R^2^ of 0.899.

Upon further analysis, we found that the prediction of TH is challenging primarily due to its relatively narrow numerical range (approximately 0.35 to 1.09 mmol/L) compared to other parameters, like, BOD, COD, and TA, which exhibit broader value ranges. This limited variation makes it difficult for the model to learn distinct patterns and, results in higher relative prediction errors. Additionally, TH appears to be influenced by complex chemical and physical factors that are not fully captured by the input features in the current dataset. The sensitivity of TH measurements to small environmental fluctuations may also contribute to increased noise, further complicating accurate prediction. This difficulty in predicting TH impacts the overall model performance, especially in metrics that average errors across all targets, as even small absolute errors in TH translate to significant relative errors due to its scale.

The MAE heatmaps provide insights into error distribution. The training MAE heatmap shows relatively low errors for BOD and COD but higher errors for NH_3_-N and TDS, suggesting that while the model captures overall trends well, there is some difficulty in reducing errors for these parameters.

**Fig. 12:**
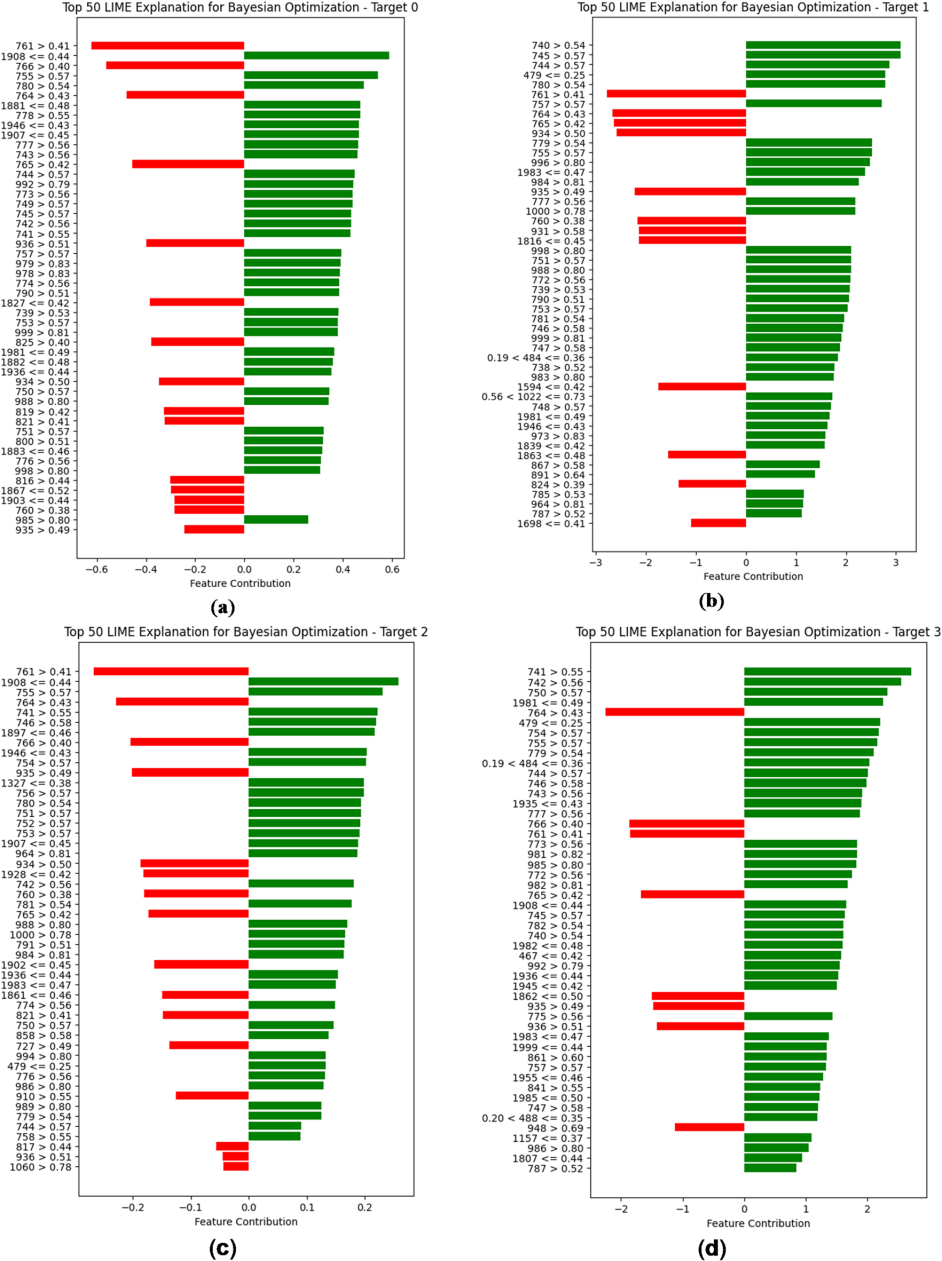

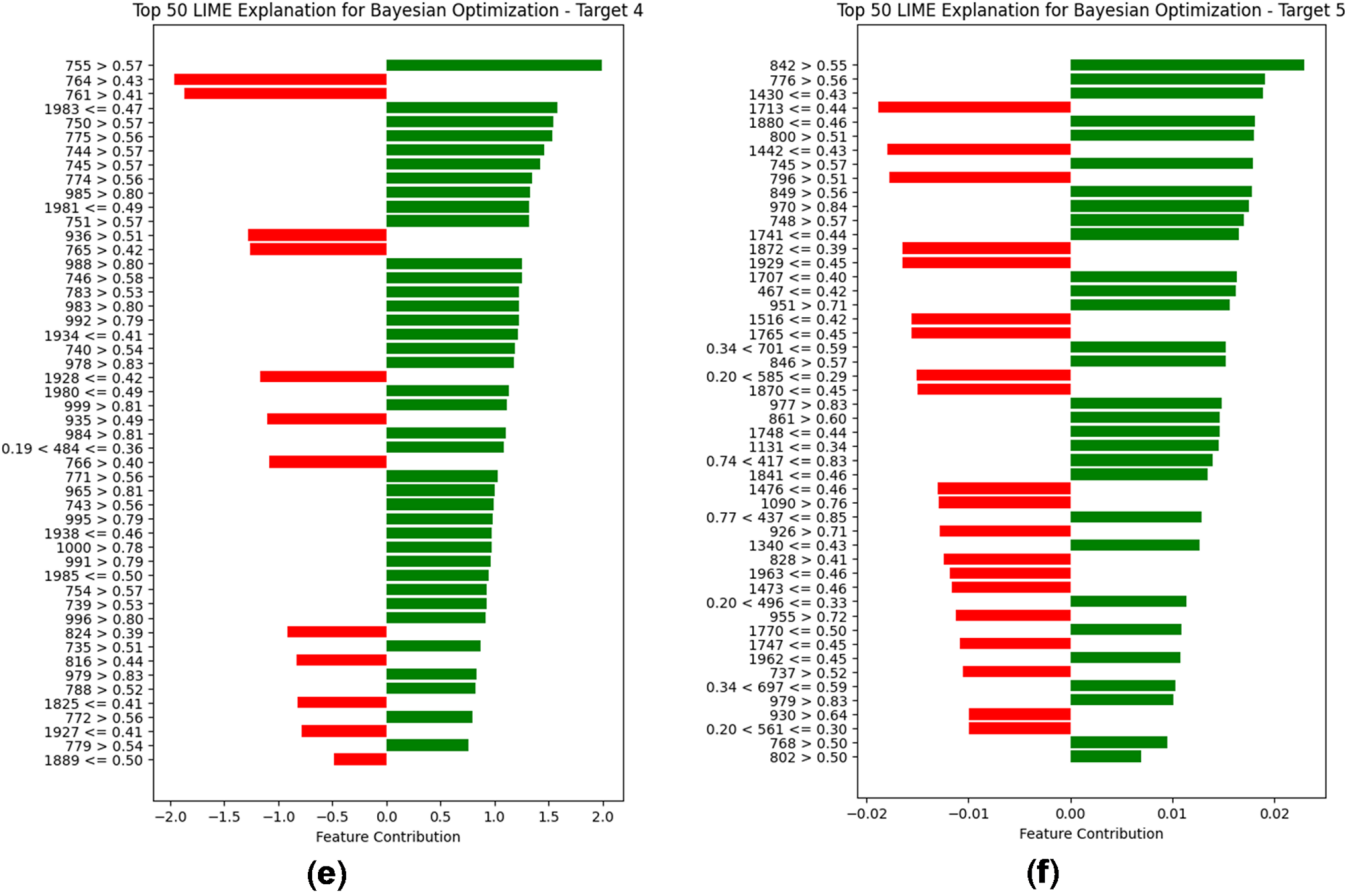
(**a**) LIME interpretation of the Bayesian Optimized Neural Network architecture for prediction of BOD. (**b**) LIME interpretation of the Bayesian Optimized Neural Network architecture for prediction of COD. (**c**) LIME interpretation of the Bayesian Optimized Neural Network architecture for prediction of NH_3_-N. (**d**) LIME interpretation of the Bayesian Optimized Neural Network architecture for prediction of TDS. (**e**) LIME interpretation of the Bayesian Optimized Neural Network architecture for prediction of TA. (**f**) LIME interpretation of the Bayesian Optimized Neural Network architecture for prediction of TH.

The comprehensive analysis shows that the architecture designed by the Bayesian Optimization algorithm performs well for multi-target predictions by predicting all six parameters with high accuracy and less MAE. Figure 12 depicts the feature importance of the optimal architecture.

The LIME analysis of predictions across all six targets highlights key feature contributions and their impact on the model’s decision-making process is depicted in Figs. 12 (a-e). For BOD, the prediction is strongly influenced by features such as 761 > 0.41 and 1908 ≤ 0.44, with a clear dominance of positive contributions, suggesting that the model confidently distinguishes this class. Similarly, TDS exhibits high stability, with features like 741 > 0.55 and 742 > 0.56 playing a significant role, contributing mostly positively and leading to a reliable prediction. In contrast, COD presents a more complex decision boundary, as both positive and negative feature influences are substantial, indicating a level of uncertainty in classification. A similar trend is observed in NH3-N, where feature contributions show overlap with Target 0 but with a more balanced distribution of positive and negative influences, potentially making predictions for this class less definitive.

For TA, the model’s confidence appears to decline slightly due to a higher number of negatively contributing features, such as 1983 ≤ 0.47, which counteract the positive impact of key features like 755 > 0.57 and 761 > 0.41. The least stable prediction is observed in TH, where negative contributions outweigh positive ones, suggesting that the model faces significant difficulty in distinguishing this class from others. Notably, features such as 842 > 0.55 and 776 > 0.56 play crucial roles, but their effectiveness is diminished by opposing influences from features like 1430 ≤ 0.41 and 1713 ≤ 0.44.

Across all targets, several features, including 755, 761, 764, and 745, appear repeatedly, indicating their significance in multiple classifications. However, the shifting impact of these features between targets suggests potential overlaps in decision boundaries, leading to occasional prediction uncertainty. Overall, while the model demonstrates confidence in some targets, particularly 0 and 3, it struggles with others, such as 5 and 1, where conflicting feature influences introduce ambiguity.

The LIME-based explanations presented in this study are not just interpretability tools for data scientists but can be directly utilized by wastewater treatment plant engineers for operational insights. For example, by identifying the top contributing features influencing predictions for critical parameters like BOD, COD, or NH_3_-N, engineers can:

- Prioritize maintenance: Features consistently contributing heavily to the prediction can guide which processes require closer monitoring or earlier maintenance.
- Implement proactive adjustments: If LIME indicates that a high TDS value consistently lowers predicted water quality, operators can proactively increase filtration or adjust coagulant doses.
- Explain model decisions to regulators: The visual and interpretable format of LIME enables transparent reporting of AI-assisted decisions, aligning with regulatory compliance needs.

In a scenario where the model predicted unusually high BOD, LIME indicated that the respective feature contributed significantly. This helped the operator identify and adjust a faulty recirculation pump, thus preventing a regulatory violation.

### Statistical analysis of hyperparameter tuning strategies

To assess the comparative performance of the four hyperparameter tuning strategies—Bayesian Optimization, Grid Search, Hyperband, and Random Search—a comprehensive statistical evaluation was conducted using non-parametric tests.

**Fig. 13:**
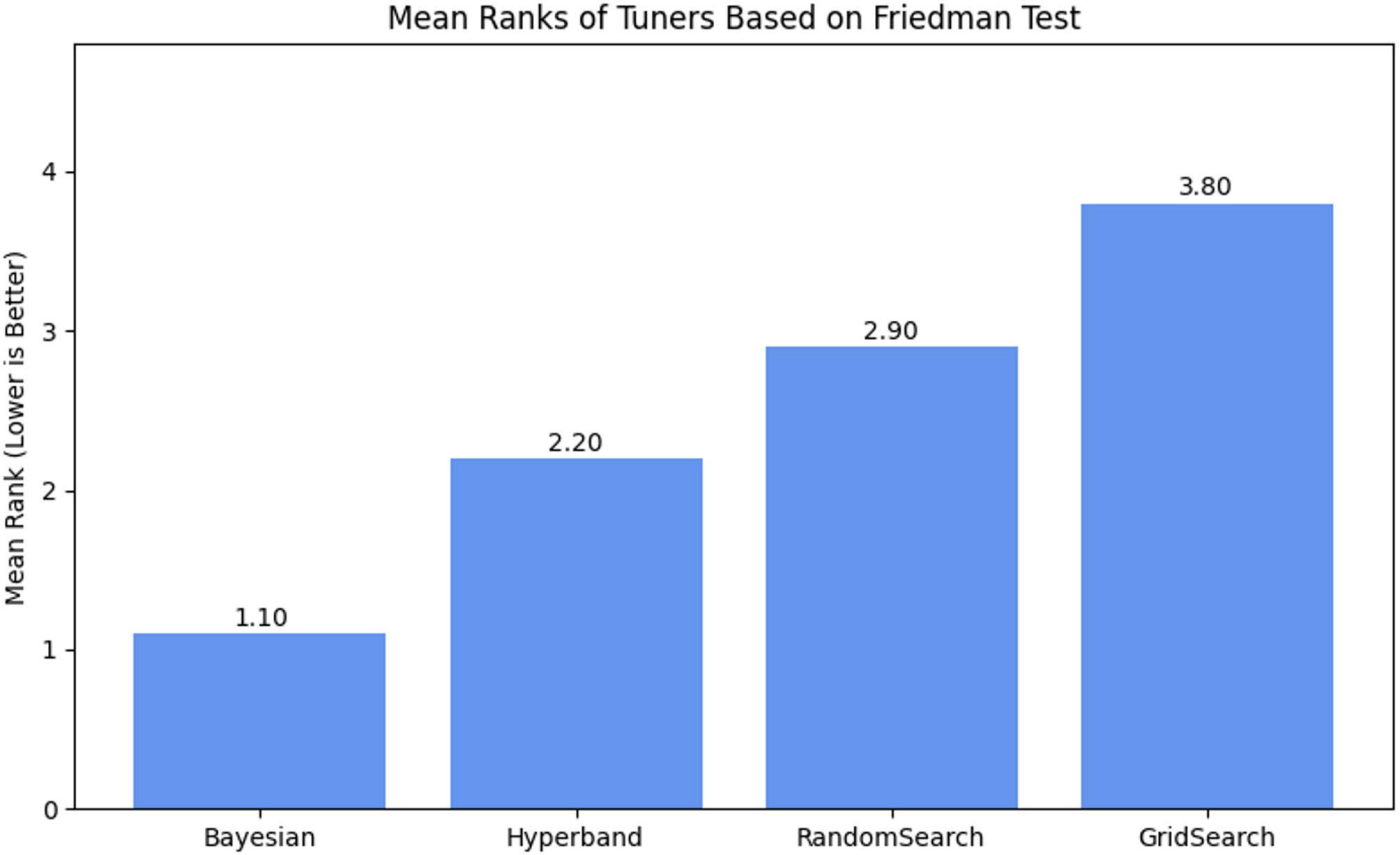
Mean ranks of tuners based on Friedman test.

First, the Friedman test was performed to determine whether the observed differences among the tuners were statistically significant. As shown in the Fig. 13 mean rank bar chart, Bayesian Optimization achieved the lowest average rank (1.10), while Grid Search had the highest (3.80). The Friedman chi-square statistic was χ^2^ = 11.9388, with a p-value = 0.0076, indicating statistically significant differences among the tuners.

To further validate this outcome, the Iman-Davenport correction was applied. This test provided a more sensitive evaluation, especially for small sample sizes. The calculated Iman-Davenport statistic was F = 15.6000, with a p-value = 0.0002, confirming the rejection of the null hypothesis that all tuning methods perform equally.

**Fig. 14:**
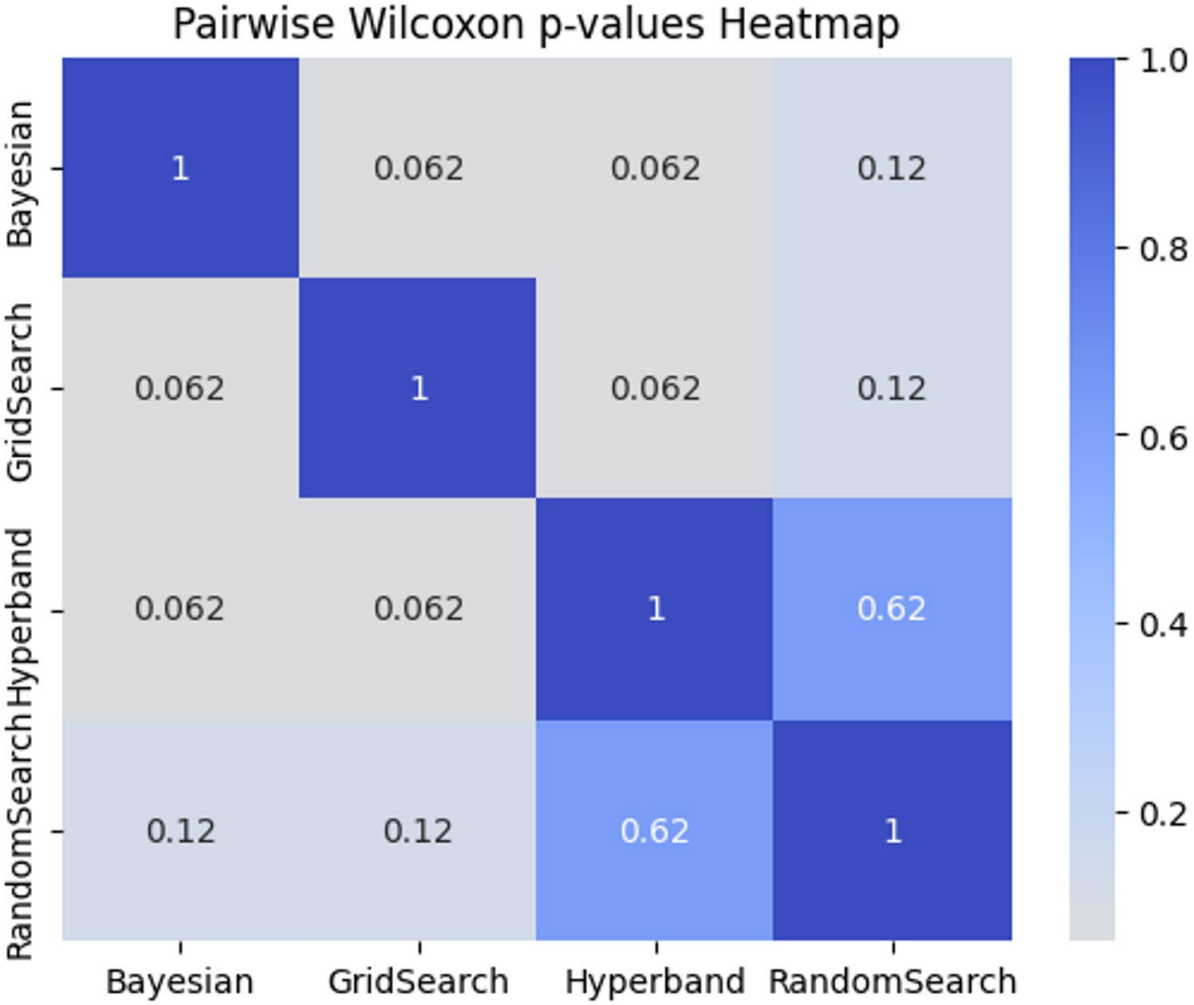
Pairwise Wilcoxon p-values Heatmap.

Following the Friedman test, post-hoc pairwise comparisons were conducted to explore differences between specific pairs of tuners:

Pairwise Wilcoxon tests were applied between all combinations of tuners. As shown in the Wilcoxon p-values heatmap in Fig. 14, none of the pairwise comparisons reached statistical significance after Bonferroni adjustment. For example, the comparison between Bayesian and Grid Search yielded a raw p-value of 0.0625, adjusted to 0.375, which is above the α = 0.05 threshold.

**Fig. 15:**
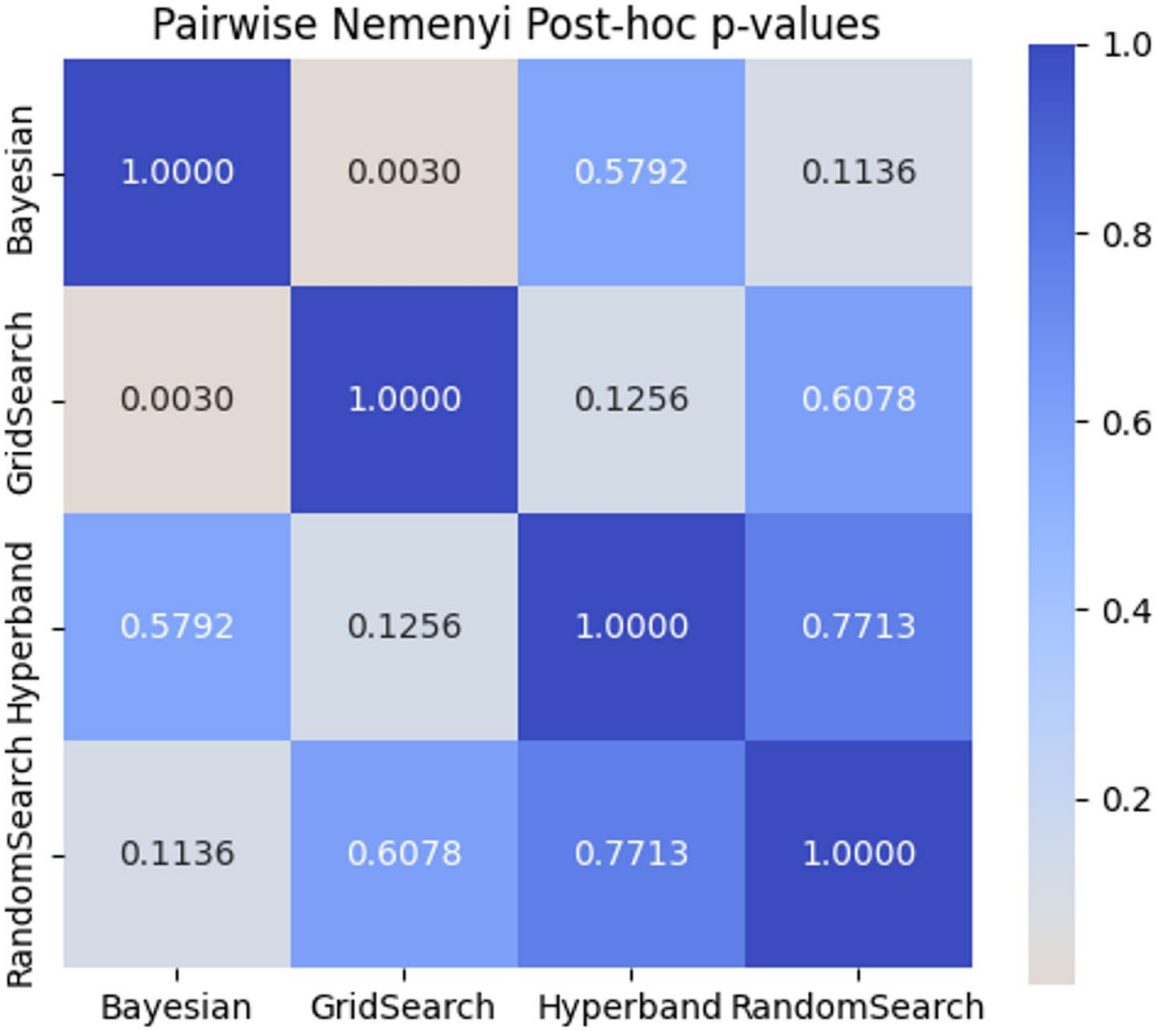
Pairwise Nemenyi p-values Heatmap.

The Nemenyi test was also conducted for all pairwise comparisons. The Nemenyi p-value heatmap as shown in Fig. 15 highlights the significance levels between each pair. The most prominent finding was between Bayesian Optimization and Grid Search, where the p-value was 0.0030, suggesting a statistically significant difference in their average ranks. However, other pairwise comparisons such as Bayesian vs. Hyperband (*p* = 0.5792) and Grid Search vs. Random Search (*p* = 0.6078) did not yield significant results.

These results indicate that while the Friedman and Iman-Davenport tests show an overall significant difference in tuner performance, the pairwise differences are not uniformly significant. The most consistent outperformer across evaluations was Bayesian Optimization, as evidenced by its lowest average rank and statistically significant superiority over Grid Search in the Nemenyi test.

The analysis supports the conclusion that Bayesian Optimization consistently outperforms the other tuning strategies, both in terms of mean rank and pairwise comparisons. While the Wilcoxon test did not yield significant pairwise results under strict correction, the Nemenyi test confirmed a meaningful difference between the top and bottom-ranked strategies, as seen in the visualizations. These results validate the reliability of the Friedman test outcome and provide strong statistical support for the selection of Bayesian methods in model tuning.

### Practical applicability in wastewater treatment plants

To demonstrate the practical applicability of the proposed approach, it is important to connect the results of this study with real-world scenarios in wastewater treatment. In operational wastewater treatment plants, accurate and timely prediction of water quality parameters such as BOD, COD, TDS, NH_3_-N, TA, and TH is critical for process optimization, chemical dosing, and compliance with environmental regulations. The use of neural networks optimized through automated search algorithms, as demonstrated in this study, allows for reliable multi-target prediction, enabling plant engineers to make informed decisions based on real-time sensor data. For instance, predicting rising COD levels could prompt early intervention to adjust aeration or chemical inputs, thereby preventing process failures. Similarly, the ability to forecast NH_3_-N concentrations can help manage nitrification processes more efficiently. Such predictive insights support proactive and adaptive management strategies, improving both operational efficiency and environmental performance. These findings align with the broader industry trend of adopting AI-based tools for smart water infrastructure, thus reinforcing the relevance of the proposed model for deployment in real-world wastewater management systems.

## Limitations

This study acknowledges the following methodological limitations:

- Dataset size and usage of a diverse dataset.
  The dataset used for training and evaluation consists of a relatively small number of samples collected from a single wastewater treatment plant. To improve the generalizability and robustness of the model, the experiment can be extended by incorporating various real-time datasets from multiple wastewater treatment plants with different operational conditions.

- Absence of Cross-Validation.
  Due to the limited size of the dataset and computational constraints, k-fold cross-validation was not applied. Instead, a fixed train-test split was used. This may introduce variance in model evaluation and limit robustness.

- Computational Limitations.
  All experiments were conducted on a CPU-based system (Intel Core i7, 16 GB RAM, no GPU). This restricted the exploration of deeper neural architectures and longer training durations.

- Limited Global Interpretability.
  Although LIME was used for local explainability, the model’s global behavior remains complex. This might pose challenges for wastewater engineers interpreting results without a strong data science background.

## Conclusion

The results of this study imply that AutoML is an effective tool for determining neural network (NN) architectures for wastewater quality prediction. Utilizing NAS with different search algorithms (such as Random Search, Grid Search, Bayesian Optimization, and Hyperband), the study successfully predicts many wastewater characteristics (BOD, COD, NH_3_-N, TDS, TA, and TH) from spectral reflectance data. Overall, for both single-target and multi-target prediction problems, Bayesian Optimization stood out as the best search method with the lowest mean absolute error (MAE) and the highest coefficient of determination (R^2^) values when compared to the other search methods tested. For six-target predictions, however, the Hyperband search showed better performance in predicting TH values.

The AutoML approach not only eliminates the need for manual neural network architecture optimization—which does not scale well across diverse wastewater datasets—but also enhances model efficiency. Neural networks contain many parameters that must be fine-tuned to achieve accurate predictions, and LIMEXAI was used to ensure the interpretability of these predictions. The practical significance of our findings is that neural networks can effectively integrate various data sources (e.g., flow regime and water quality) to enable AutoML-based real-time decision-making in wastewater treatment plants.

However, the prediction of TH remains relatively more challenging than other parameters due to its narrow value range and complex dependency on unobserved or weakly correlated features. This leads to higher relative prediction errors. Addressing this challenge will be a key focus of our future work, where we plan to explore more advanced feature engineering, domain-specific inputs, and hybrid modeling approaches to enhance TH prediction accuracy and robustness.

## Data Availability

All data used to support the findings of this study are included within the article.
